# Lifespan development of EEG alpha and aperiodic component sources is shaped by the connectome and axonal delays

**DOI:** 10.1093/nsr/nwag076

**Published:** 2026-02-06

**Authors:** Ronaldo Garcia Reyes, Ariosky Areces Gonzalez, Ying Wang, Yu Jin, Shahwar Yasir, Maria Luisa Bringas-Vega, Mitchell Valdes-Sosa, Cheng Luo, Peng Xu, Viktor Jirsa, Dezhong Yao, Ludovico Minati, Pedro A Valdes-Sosa

**Affiliations:** Clinical Hospital of Chengdu Brain Science Institute, University of Electronic Science and Technology of China, Chengdu 610054, China; Neuroinformatics, Cuban Neurosciences Center, Havana 11300, Cuba; China-Cuba Belt and Road Joint Laboratory on Neurotechnology and Brain-Apparatus Communication, University of Electronic Science and Technology of China, Chengdu 610054, China; Clinical Hospital of Chengdu Brain Science Institute, University of Electronic Science and Technology of China, Chengdu 610054, China; China-Cuba Belt and Road Joint Laboratory on Neurotechnology and Brain-Apparatus Communication, University of Electronic Science and Technology of China, Chengdu 610054, China; School of Technical Sciences, University “Hermanos Saiz Montes de Oca” of Pinar del Río, Pinar del Río 20100, Cuba; Clinical Hospital of Chengdu Brain Science Institute, University of Electronic Science and Technology of China, Chengdu 610054, China; China-Cuba Belt and Road Joint Laboratory on Neurotechnology and Brain-Apparatus Communication, University of Electronic Science and Technology of China, Chengdu 610054, China; Clinical Hospital of Chengdu Brain Science Institute, University of Electronic Science and Technology of China, Chengdu 610054, China; China-Cuba Belt and Road Joint Laboratory on Neurotechnology and Brain-Apparatus Communication, University of Electronic Science and Technology of China, Chengdu 610054, China; Clinical Hospital of Chengdu Brain Science Institute, University of Electronic Science and Technology of China, Chengdu 610054, China; China-Cuba Belt and Road Joint Laboratory on Neurotechnology and Brain-Apparatus Communication, University of Electronic Science and Technology of China, Chengdu 610054, China; Clinical Hospital of Chengdu Brain Science Institute, University of Electronic Science and Technology of China, Chengdu 610054, China; China-Cuba Belt and Road Joint Laboratory on Neurotechnology and Brain-Apparatus Communication, University of Electronic Science and Technology of China, Chengdu 610054, China; Neuroinformatics, Cuban Neurosciences Center, Havana 11300, Cuba; China-Cuba Belt and Road Joint Laboratory on Neurotechnology and Brain-Apparatus Communication, University of Electronic Science and Technology of China, Chengdu 610054, China; Clinical Hospital of Chengdu Brain Science Institute, University of Electronic Science and Technology of China, Chengdu 610054, China; China-Cuba Belt and Road Joint Laboratory on Neurotechnology and Brain-Apparatus Communication, University of Electronic Science and Technology of China, Chengdu 610054, China; Laboratory for Brain Science and Artificial Intelligence, Southwest University of Science and Technology, Mianyang 621010, China; Aix Marseille Université, Institut National de la Santé et de la Recherche Médicale, Institut de Neurosciences des Systèmes (INS) UMR1106; Marseille 13005, France; Clinical Hospital of Chengdu Brain Science Institute, University of Electronic Science and Technology of China, Chengdu 610054, China; China-Cuba Belt and Road Joint Laboratory on Neurotechnology and Brain-Apparatus Communication, University of Electronic Science and Technology of China, Chengdu 610054, China; Clinical Hospital of Chengdu Brain Science Institute, University of Electronic Science and Technology of China, Chengdu 610054, China; China-Cuba Belt and Road Joint Laboratory on Neurotechnology and Brain-Apparatus Communication, University of Electronic Science and Technology of China, Chengdu 610054, China; Center for Mind/Brain Science (CIMeC), University of Trento, Trento 38123, Italy; Clinical Hospital of Chengdu Brain Science Institute, University of Electronic Science and Technology of China, Chengdu 610054, China; Neuroinformatics, Cuban Neurosciences Center, Havana 11300, Cuba; China-Cuba Belt and Road Joint Laboratory on Neurotechnology and Brain-Apparatus Communication, University of Electronic Science and Technology of China, Chengdu 610054, China

**Keywords:** lifespan, spectral component, EEG dataset, conduction delay, source analysis, alpha rhythm

## Abstract

We introduce ${\xi }$–${\alpha }$NET, a model of cortical activity that represents EEG aperiodic ($\xi$) and $\alpha$-rhythm ($\alpha$) components as Hida–Matérn processes constrained by anatomical connectivity and interareal conduction delays. This approach integrates the decomposition of spectral Granger causality and quantifies the lifespan trajectories of spectral processes. Using Bayesian inversion on cross-spectral rsEEG data from 1965 participants aged 5–100 (HarMNqEEG dataset), the model estimates cortical activity with high test-retest reliability, effective connectivity patterns and conduction delays. Given the approximate cortical hierarchy inferred from the inverted T1w/T2w myelination map, used as a proxy for feedforward and feedback organization, the aperiodic and ${\alpha }$ components reveal opposite directional networks across the lifespan: the aperiodic component is localized in the frontal cortex, whereas the ${\alpha }$ component is localized in the posterior cortex, with feedforward and feedback-directed connections, respectively. For both processes, the spectral parameters follow a nonlinear inverted U-shape lifespan trajectory. Finally, the model uniquely estimates global conduction delays, which are negatively correlated with $\alpha$ frequency and with independent cortical myelination (T1w/T2w) measures, consistent with a mechanistic link between conduction delays and $\alpha$-rhythm modulation.

## Teaser

This paper introduces Xi-AlphaNET, a connectome-constrained cortical EEG model that separates alpha and aperiodic processes, reveals their opposing directed networks across the lifespan, captures nonlinear developmental trajectories, and links peak alpha frequency to myelin-dependent conduction delays.

## INTRODUCTION

Spectral component models (SCMs) have become a standard framework for decomposing neural oscillations into meaningful constituents. In Table 1, we present a systematic review of the main SCMs in the literature. In this paper, we analyze neural oscillations using the cross-spectrum, defined from an EEG vector time series $\mathbf {v}_t \in \mathbb {R}^{N_c \times 1}$:


(1) \begin{eqnarray*}
{\bf\Sigma }_\omega = \mathbb {E}[\mathbf {v}_\omega \mathbf {v}_\omega ^{\dagger }] \in \mathbb {R}^{N_c \times N_c}.
\end{eqnarray*}

Here $N_c$ is the number of sensors, $\mathbb {E}$ denotes the expectation over time or trials, $\mathbf {v}_\omega$ represents the Fourier transform of the EEG signal, the symbol $(\cdot )^\dagger$ denotes conjugate transpose and $\bf{\Sigma }_\omega$ denotes the cross-spectrum at frequency $\omega$. The diagonal elements $\bf{\Sigma }_{\omega , ii}$ capture the power spectra of individual channels, while the off-diagonal elements reveal channel interactions.

**Table 1.** Comparison of the main spectral component models (SCMs). All SCMs approximate the aperiodic component as a Matérn process or one of its limiting cases.

	Component distribution									
Model	AC	α-peak	Others	Scale	Cross	Nonstat.	Anat. priors	ESI	Connectivity	Ref.
Zetterberg	AR(1)	AR(2)	–	1	–	–	–	–	–	[5]
$\xi$ –$\alpha$	$\mathcal {M}$	$\mathcal {HM}$	–	1	–	–	–	–	–	–
Multi $\xi$–$\alpha$	$\mathcal {M}$	$\mathcal {HM}$	–	1	$\checkmark$	–	–	–	–	[1]
Dipolar $\xi$–$\alpha$	$\mathcal {M}$	$\mathcal {HM}$	–	1	$\checkmark$	–	–	Dipolar	Functional	[12]
BOSC	$\mathcal {M}$	$\chi ^2$	$\chi ^2$	log-log	–	–	–	–	–	[7]
IRASA	$\mathcal {M}$	–	–	log-log	–	$\checkmark$	–	–	–	[8]
FOOOF	$\mathcal {M}$	$\mathcal {N}$	$\mathcal {N}$	log	–	$\checkmark$	–	MNE	–	[2]
SPRiNT	$\mathcal {M}$	$\mathcal {N}$	$\mathcal {N}$	log	–	$\checkmark$	–	–	–	[9]
PAPTO	$\mathcal {M}$	$\mathcal {N}$	$\mathcal {N}$	log	–	$\checkmark$	–	–	–	[10]
$\xi$ –$\pi$	Monotonic	Unimodal	Unimodal	1	–	–	–	–	–	[11]
Cortical $\xi$–$\alpha$	$\mathcal {M}$	$\mathcal {HM}$	–	1	$\checkmark$	–	–	eLORETA	Functional	[14]
$\xi$ –$\alpha$NET	MVAR + $\mathcal {M}$	MVAR + $\mathcal {HM}$	–	1	$\checkmark$	–	$\checkmark$	Bayesian	Effective	–

Distribution: statistical distribution assumed for each component—AR, autoregressive model; $\mathcal {M}$, Matérn process; $\mathcal {HM}$, Hida–Matérn process; $\mathcal {N}$, Gaussian (normal); $\chi ^2$, chi-squared; Monotonic, constrained monotonic shape; Unimodal, single-peak shape. Scale: frequency axis scaling—1, linear; log, logarithmic; log-log, log-power versus log-frequency. Cross: multivariate modeling of the cross-spectra. Nonstat.: nonstationary modeling capability. Anat. priors: use of structural priors, such as connectome and delays. ESI: type of electrophysiological source imaging used (e.g. MNE, eLORETA, Bayesian). Connectivity: Functional, undirected association; Effective, directed or causal influence models.

**Figure 1.**
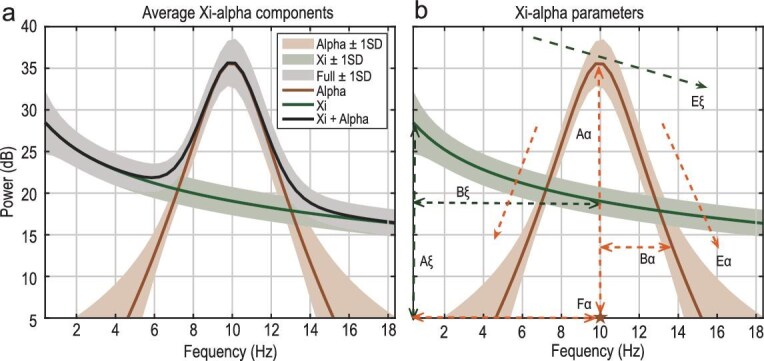
$\xi$ –$\alpha$ spectral decomposition for a representative subject. (a) The empirical EEG power spectrum (gray band) is modeled as a linear superposition of two physiologically interpretable and independent components with generalized Lorentz spectral profiles. The first component, the aperiodic process $\xi$ (green line), captures the characteristic $1/\omega$ spectral decay. The second component, the $\alpha$ process (orange line), models narrowband oscillatory activity centered within the 7–13 Hz range, corresponding to $\alpha$ rhythms. The combined $\xi$–$\alpha$ model (black line) accurately captures the empirical spectral shape by summing the independent contributions of both components. Shaded regions indicate $\pm 1$ standard deviation across all EEG sensors for a subject. (b) The components are modeled at each sensor *i* using a generalized Lorentz spectral distribution given by $\psi (\omega \mid A,B,E,F)=A/(1+B(\omega -F)^2)^E$, where *A* is the amplitude (in decibels), *B* is the bandwidth (in s$^2$), *E* is the spectral exponent and *F* is the frequency shift (in hertz). The aperiodic component is defined as $\xi _{\omega ,i}=\psi (\omega \mid A\xi _i,B\xi _i,E\xi _i,0)$, and the $\alpha$ spectral peak as $\alpha _{\omega ,i}=(\psi (\omega \mid A\alpha _i,B\alpha _i,E\alpha _i,F\alpha _i)+\psi (\omega \mid A\alpha _i,B\alpha _i,E\alpha _i,-F\alpha _i))/2$. Here, $A\xi _i$ and $A\alpha _i$ quantify the amplitude of each component; $B\xi _i$ and $B\alpha _i$ control spectral bandwidth; $E\xi _i$ and $E\alpha _i$ determine spectral decay; and $F\alpha _i$, indicated by the orange star on the frequency axis, denotes the peak alpha frequency (PAF) and characterizes the modal frequency of the $\alpha$ rhythms [6].

Across the literature, two key spectral components (SCs) consistently emerge (Fig. 1): the aperiodic component ($\xi$ process), characterized by a monotonic decay with frequency, and the periodic component, with a notable resonant peak in the $\alpha$ band (7–13 Hz), known as the $\alpha$ process [1–4]. The $\xi$ process is sometimes approximated by a $1/\omega ^\beta$ decay, corresponding to a fractional Brownian process in the time domain.

Two complementary approaches have historically shaped SCM development (Table 1). The foundational work of Zetterberg *et al.* [5] employed a time-domain approach using autoregressive moving average (ARMA) models to estimate EEG spectra. In contrast, Pascual-Marqui *et al.* [1] pioneered a frequency-domain approach with the $\xi$–$\alpha$ model, representing EEG spectra as a mixture of independent stochastic processes: the $\xi$ and $\alpha$ processes. Pascual-Marqui’s adoption of a generalized Lorentzian profile—originally inspired by spectroscopy—enabled efficient fitting to EEG spectra (see Fig. 1). Subsequent work by Amador *et al.* [6] demonstrated the success of this model in capturing developmental changes. As we show, the Lorentzian spectral profiles that characterize the $\xi$–$\alpha$ model arise from time-domain processes governed by the Hida–Matérn class. This connection extends beyond the aperiodic component, suggesting that all EEG components are driven by fractional stochastic dynamics—a viewpoint largely absent in contemporary SCMs.

Recent SCMs have diversified, adopting the frequency-domain framework introduced by Pascual-Marqui through the $\xi$–$\alpha$ model (Table 1). Univariate SCMs, such as BOSC [7], IRASA [8], FOOOF [2], SPRiNT [9], PAPTO [10] and the recent $\xi$–$\pi$ model [11], offer a range of parametric and non-parametric techniques. In parallel, multivariate approaches have been developed to model the off-diagonal elements of the cross-spectra. An early example is the multivariate $\xi$–$\alpha$ model[1], although it is restricted to sensor-level EEG. Valdés-Sosa *et al.* [12] later proposed the first source-level formulation, modeling $\xi$ as an isotropic process on the cortical surface and $\alpha$ rhythms as two correlated stochastic dipole processes with fixed orientations and magnitudes. Jirsa *et al.* [13] demonstrated that spatially extended neural fields with homogeneous connectivity and embedded heterogeneous connections can similarly explain $\xi$–$\alpha$ processes. In both cases, the rationale is the same: isotropically distributed sources are causally linked to $\xi$, whereas more localized sources are linked to $\alpha$. More recently, Pascual-Marqui *et al.* [14] introduced cortical $\xi$–$\alpha$, which extends the $\xi$–$\alpha$ model to cortical source space using eLORETA for spectral functional connectivity estimation.

As we can conclude from Table 1, current approaches to estimating the neural sources of SCs typically follow a two-step strategy: (i) localizing activity to putative source sensors, followed by (ii) fitting SCM parameters. These methods neglect key anatomical and functional constraints, such as structural connectivity and conduction delays, that shape the spatiotemporal propagation of oscillatory activity [15,16]. As we have previously shown, such a two-step strategy can lead to biased estimates due to misspecified source covariance matrices [17].

Solutions to these challenges can be found in large-scale initiatives such as the Digital Twin Brain and the Virtual Brain Project [18,19], which integrate rich neuroanatomical data to capture neural oscillations more realistically. However, these approaches require substantial computational resources, limiting their direct practical applicability in large-scale normative studies. To overcome these limitations, we introduce $\xi$–$\alpha$NET, a mesoscale generative model of cortical activity formulated as a sparse, structurally constrained network of Hida–Matérn processes. Unlike previous SCM approaches, $\xi$–$\alpha$NET enables the joint inference of source-level spectral dynamics and effective connectivity across the lifespan.

## RESULTS

### $\xi$ –$\alpha$NET as a sparse structurally constrained network of the Hida–Matérn process

#### Forward model

We propose $\xi$–$\alpha$NET, a mesoscale generative model of cortical dynamics that accounts for EEG observations by integrating vertex-wise spectral processes with anatomically grounded structural connectivity and conduction delays. Specifically, the observed EEG time series $\mathbf {v}_t \in \mathbb {R}^{N_c \times 1}$ is modeled as a linear projection of distributed cortical activity:


(2) \begin{eqnarray*}
\mathbf {v}_t = \mathbf {K} \mathbf {j}_t + \mathbf {n}_t,
\end{eqnarray*}

where $\mathbf {K} \in \mathbb {R}^{N_c\times N_v}$ is the lead-field matrix and $\mathbf {n}_t$ is zero-mean Gaussian noise. We aim to model the time-domain cortical activity $\mathbf {j}_t \in \mathbb {R}^{N_v}$ at each thalamocortical unit under three constraints:

- the model must incorporate structural priors, reflecting structural connectivity and conduction delays across neurotracts, which shape the spatiotemporal correlations in a causal manner;
- the spectral dynamics must arise as a linear superposition of two statistically independent components, corresponding to aperiodic and oscillatory generators;
- the resulting power spectrum must exhibit Lorentzian profiles, consistent with empirical spectra observed across multiple frequency bands in EEG/MEG recordings.

To satisfy condition (i), we model cortical dynamics within a structurally constrained multivariate autoregressive (MVAR) network, in which the autoregressive coefficients are informed by structural priors. However, simple MVAR or AR models fail to reproduce the full characteristics of Lorentzian spectral profiles of neural oscillations, particularly in the $\alpha$ band. Therefore, it is necessary to employ MVAR models with colored innovations.

Condition (ii) is satisfied if we assume that cortical activity $\mathbf {j}_t$ is a linear superposition of two independent stochastic processes modelling the activations of independent SCs. This approach is aligned with the linear decomposition of the cross-spectrum in SCs used in multivariate $\xi$–$\alpha$ models proposed by Pascual-Marqui *et al.* [1,12]. However, despite the success of $\xi$–$\alpha$ models, the underlying time-domain mechanisms capable of generating Lorentzian spectral profiles have not previously been explained. Here, we present a formulation that addresses this gap: *Lorentzian spectral profiles can be generated by real-valued fractional stochastic processes belonging to the Matérn class*. These are Gaussian processes with a temporally delayed structure or autocorrelation. Matérn processes have been widely used in spatial statistics and machine learning; however, their application to modelling cortical time series has been overlooked in the neuroscience community. As shown in Table 1, we find that the aperiodic component of neural oscillation can be explained in terms of a Matérn process, providing a principled time-domain origin of the $\xi$ process defined by Pascual-Marqui *et al.* [1,12].

Resonant spectral peaks, such as those observed in the $\alpha$ process or other rhythms, present an additional challenge because they reflect intrinsic oscillatory dynamics that conventional Matérn processes alone cannot fully capture. Early work, such as the thalamocortical Robinson model [20,21], demonstrated that damped differential equations can generate oscillatory modes through the interplay of damping and delays. However, the Robinson model cannot reproduce the full range of spectral patterns observed in the $\alpha$ band, particularly the Lorentzian spectral profiles with arbitrary exponents, which govern the smoothness of the corresponding time-domain processes introduced by Pascual-Marqui *et al.* [1,12,14]. Properly modeling of these features requires the use of fractional stochastic differential equations, as implemented in oscillatory Matérn processes [22].

Although oscillatory Matérn processes introduce a resonant spectral peak, their time-domain representation requires complex-valued stochastic processes [22]. This is inconsistent with the real-valued nature of neurophysiological signals. Therefore, in order to address this limitation, we model the $\alpha$ rhythm within each thalamocortical unit using the Hida–Matérn process. Unlike the oscillatory Matérn process, the Hida–Matérn formulation yields an analytically tractable, physically interpretable and real-valued process in a time-domain model that aligns with the expected empirical properties of cortical activity [23]. Importantly, Hida–Matérn processes are exact solutions to fractional stochastic differential equations. This mathematical framework has been successfully applied to complex phenomena such as turbulence and anomalous diffusion, and is extended here, through our model, to neural oscillations.

**Figure 2.**
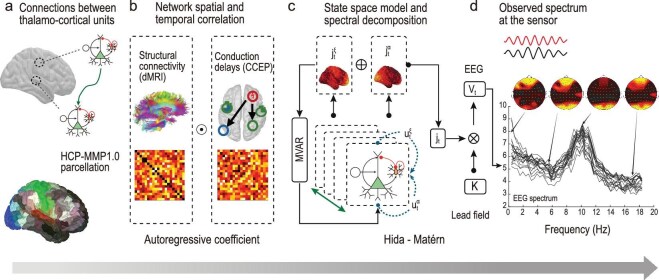
$\xi$ –$\alpha$NET forward model. (a) Connectivity between cortical generators is constrained using structural connectivity from diffusion MRI (dMRI) tractography and conduction delays estimated from cortico-cortical evoked potentials (CCEPs), with the HCP-MMP1 parcellation [24] used to define the cortical generators. (b) Structural connectivity and conduction-delay estimates shape the baseline spatiotemporal correlations of the cortical network. (c) Cortical activity ($\mathbf {j}_t$) arises from the linear superposition of two independent spectral processes, where $\mathbf {j}^\xi _t$ accounts for the aperiodic component and $\mathbf {j}^\alpha _t$ for the $\alpha$ spectral peak. Each component is modeled using an independent MVAR state-space model with colored Hida–Matern innovations $\mathbf {u}^{\xi }_t$ and $\mathbf {u}^{\alpha }_t$. The autoregressive coefficients of the MVAR model are shaped by the connectome and conduction delays, allowing for non-local interactions between cortical generators, while the independent Hida–Matern innovations represent local processes at each thalamocortical unit. This formulation enables the introduction of Lorenzian spectral profiles for each spectral process, with parameters that have a sparse distribution over the cortical surface. (d) Cortical activity is then projected to the sensors using the lead-field matrix ($\mathbf {K}$), yielding observed EEG spectra consistent with electrophysiological data. This framework enables the estimation of cortical SCs, conduction delays and source effective connectivity from EEG/MEG recordings, supporting large-scale normative studies of cortical maturation. The symbol ‘$\odot$’ denotes the Hadamard (element-wise) product used to introduce a spatiotemporal decomposition of interactions between generators of neural activity; ‘$\oplus$’ denotes summation and ‘$\otimes$’ denotes the standard matrix product.

Accordingly, one possible linear model of cortical activity that satisfies all three conditions can be expressed using an MVAR model with colored innovations as (Fig. 2)


(3) \begin{eqnarray*}
\mathbf {j}_t &=& \mathbf {j}_t^\xi + \mathbf {j}_t^\alpha ,\nonumber\\
\mathbf {j}_t^\xi &=& (\mathbf {A} \circledast \mathbf {j}^\xi )_t + \mathbf {u}_t^\xi ,\nonumber\\
\mathbf {j}_t^\alpha &=& (\mathbf {A} \circledast \mathbf {j}^\alpha )_t + \mathbf {u}_t^\alpha ,
\end{eqnarray*}

where $(\mathbf {A} \circledast \mathbf {j})_t = \int _0^\infty \mathbf {A}_\tau \mathbf {j}_{t - \tau }\, \mathrm{d}\tau$ denotes delayed convolution over interactions. The kernel $\mathbf {A}_\tau \in \mathbb {R}^{N_v \times N_v}$ is a delay-dependent matrix of autoregressive coefficients with a null diagonal, governing nonlocal spatiotemporal propagation between thalamocortical generators. The concatenation of these delay-dependent interactions forms the delayed connectome tensor [25–27], which reflects the joint influence of anatomical connectivity and conduction delays. Petkoski and Jirsa [15] showed that incorporating delays qualitatively alters network dynamics, necessitating normalization of graph-theoretical metrics. To embed structural priors, we parametrize the convolution kernel through a spatiotemporal decomposition of interactions using a Hadamard product [25,28]:


(4) \begin{eqnarray*}
\mathbf {A}_\tau = \mathbf {C} \odot \delta (\tau - \mathbf {D}).
\end{eqnarray*}

Here $\delta (\cdot )$ is the Dirac delta function applied element-wise, and ‘$\odot$’ denotes the Hadamard (element-wise) product. The matrices $\mathbf {C} = w_C\, \bar{\mathbf {C}}$ and $\mathbf {D} = w_D\, \bar{\mathbf {D}}$ encode subject-specific coupling strengths and conduction delays, scaled from population-level priors $\bar{\mathbf {C}}$ and $\bar{\mathbf {D}} \in \mathbb {R}^{N_v\times N_v}$. The parameters $w_D$ and $w_C$ are structural modulatory weights selected within the search space to ensure that cortical activity represents a weakly stationary process and that conduction delays remain within physiologically plausible ranges, respectively. This formulation links anatomical connectivity to dynamical interactions, in line with the framework proposed by Fukushima *et al.* [29]. The driven colored Gaussian noise process $\mathbf {u}_t^\eta \in \mathbb {R}^{N_v}$, with $\eta \in \lbrace \xi , \alpha \rbrace$, models the within-thalamocortical unit dynamics for each spectral component as a collection of vertex-wise independent Hida–Matérn processes. Each process is zero mean and temporally stationary, with autocovariance structure given by


\begin{eqnarray*}
\mathbb {E}[\mathbf {u}_{t,i}^\eta \mathbf {u}_{t-\tau ,k}^{\eta ^{\prime }}] &=& \delta _{i,k} \delta _{\eta ,\eta ^{\prime }} \cos (2\pi F_i \tau ) \mathcal {M}_i(\tau ), \\
\mathcal {M}_i(\tau ) &=& \frac{2 \sqrt{\pi } A_i}{\sqrt{B_i} \Gamma (E_i)} \bigg ( \frac{\pi |\tau |}{\sqrt{B_i}} \bigg )^{E_i - {1}/{2}}\\
&&\times \, K_{E_i - {1}/{2}} \bigg ( \frac{2\pi |\tau |}{\sqrt{B_i}} \bigg ),
\end{eqnarray*}

where $\delta _{i,k}$ and $\delta _{\eta ,\eta ^{\prime }}$ are Kronecker deltas enforcing independence across vertices and SCs, respectively (not to be confused with the Dirac delta function $\delta (\tau )$ used to describe autoregressive coefficients in the convolutional model). The function $\mathcal {M}_i(\tau )$ denotes the Matérn kernel shaping the temporal envelope. Here, $A_i > 0$ is the amplitude, $B_i > 0$ controls the temporal bandwidth (inversely related to peak sharpness), $E_i > \frac{1}{2}$ determines the smoothness and $F_i \ge 0$ specifies the central frequency of oscillation. We denote by $K_\nu (\cdot )$ the modified Bessel function of the second kind, and by $\Gamma (\cdot )$ the Euler gamma function [23].

As a result of this formulation, the corresponding power spectral densities of the aperiodic and $\alpha$ components at vertex *i*, $\mathbf {u}_{t, i}^\xi$ and $\mathbf {u}_{t,i}^\alpha$, coincide with the spectrum parameterizations $\bf{\xi }_{\omega ,i}$ and $\bf{\alpha }_{\omega ,i}$ introduced by Pascual-Marqui *et al.* [1,14]:


(5) \begin{eqnarray*}
{\xi }_{\omega ,i}&=& \psi (\omega \mid A\xi _i, B\xi _i, E\xi _i, 0),\nonumber \\
{\alpha }_{\omega ,i} &=& \frac{1}{2} \psi (\omega \mid A\alpha _i, B\alpha _i, E\alpha _i, F\alpha _i )\nonumber \\
&& + \frac{1}{2} \psi (\omega \mid A\alpha _i, B\alpha _i, E\alpha _i,-F\alpha _i ),
\end{eqnarray*}

where the function $\psi (\omega \mid A, B, E, F)$ denotes a generalized Lorentzian spectral peak centered at frequency *F*, with amplitude *A*, bandwidth parameter *B* and spectral exponent *E* (Section S3 ). These parameters fully determine the shape and scale of each generator’s spectral profile (Fig. 1). Throughout this paper, we refer to these quantities as the model’s spectral parameters. In order to enforce sparsity over the SCs, we will assume group Lasso priors across the parameters of each cortical generator. This ensures that, if a given cortical generator does not contribute to a particular spectral component power (e.g. $\alpha$ or aperiodic), all corresponding spectral parameters are simultaneously driven to zero for that generator [30].

As a consequence of this formulation, the $\xi$–$\alpha$NET is a sparse, structurally constrained generative model of cortical activity in which each cortical vertex represents a lumped thalamocortical generator governed by coupled Hida–Matérn processes. Local dynamics are specified by the spectral parameters $\mathbf {A}\bf{\xi }$, $\mathbf {B}\bf{\xi }$, $\mathbf {E}\bf{\xi }$, $\mathbf {A}\bf{\alpha }$, $\mathbf {B}\bf{\alpha }$, $\mathbf {E}\bf{\alpha }$ and $\mathbf {F}\bf{\alpha } \in \mathbb {R}^{N_v\times 1}$, which characterize the aperiodic ($\xi$) and oscillatory ($\alpha$) components of the local power spectrum and implicitly capture thalamocortical dynamics. Long-range interactions are constrained by the structural parameters $w_C$ and $w_D$, which modulate the strength and delays of cortico-cortical coupling. The model does not include an explicit thalamic representation; thalamic influences are embedded locally within the spectral parameters, while connectivity is exclusively cortico-cortical. Full mathematical details are provided in Sections S1– S3.

#### Spectral Granger causality

Within the $\xi$–$\alpha$NET framework, the cortical activity of each spectral component is modeled as an independent MVAR process with Hida–Matérn innovations. This formulation implies that each spectral process defines an independent causal structure between cortical generators. Consequently, *spectral decomposition naturally entails a decomposition of effective connectivity*, in line with the decomposition of functional connectivity obtained using lagged coherence by Pascual-Marqui *et al.* [14].

However, mapping effective connectivity within the $\xi$–$\alpha$NET model cannot be performed naively using standard measures such as isolated effective coherence (iCoh), lagged coherence, spectral Granger causality (SGC), partial directed coherence (PDC) or the noise contribution ratio (NCR) [16,31–34]. These connectivity measures assume temporally uncorrelated (white) innovations within the generative model. Therefore, in order to apply them to map effective connectivity, it is necessary to express them in terms of the system transfer function (TF) of the generative model in its innovation form, or Wold representation [31,33]. In the case of the $\xi$–$\alpha$NET model, each spectral component yields a frequency-resolved transfer function with explicit parametric expressions:


\begin{eqnarray*}
\mathbf {H}^\xi _{\omega ,ik} = (\mathbf {I} - \mathbf {C} \odot \exp (-2\pi i \omega \mathbf {D}))^{-1}_{ik} \sqrt{{\xi }_{\omega ,k}}, \\
\mathbf {H}^\alpha _{\omega ,ik} = (\mathbf {I} - \mathbf {C} \odot \exp (-2\pi i \omega \mathbf {D}))^{-1}_{ik} \sqrt{{\alpha }_{\omega ,k}}.
\end{eqnarray*}

Here $\mathbf {A}_\omega = \mathbf {C} \odot \exp (-2\pi i \omega \mathbf {D})$ denotes the frequency-resolved autoregressive coefficient matrix, incorporating structural connectivity and conduction delays. From these expressions, several key conclusions emerge. First, for directed propagation to occur from a source generator *k* to a target generator *i*, the spectral process must be active at the source generator (i.e. ${\xi }_{\omega ,k}$ or ${\alpha }_{\omega ,k}$ is nonzero), and an anatomical path must exist between the source and target generators (i.e. $\mathbf {C}_{ik} \ne 0$). If ${\xi }_{\omega ,k}$ or ${\alpha }_{\omega ,k} \rightarrow 0$, propagation is effectively suppressed. Second, each spectral process also defines a distinct cross-spectrum:


(6) \begin{eqnarray*}
\mathbf {S}^\xi _\omega = \mathbf {H}^\xi _\omega \left(\mathbf {H}^\xi _\omega \right)^{\dagger },
\end{eqnarray*}

(7) \begin{eqnarray*}
\mathbf {S}^\alpha _\omega = \mathbf {H}^\alpha _\omega \left(\mathbf {H}^\alpha _\omega \right)^{\dagger }.
\end{eqnarray*}

From these spectral factorizations of the cross-spectra, we can compute the SGC to approximate the effective connectivity, as shown by Friston *et al.* [33] and Dhamala *et al.* [35], separately for each spectral process. By manipulating the cross-spectra of each spectral process, we derive an expression for the spectral power density at each generator:


(8) \begin{eqnarray*}
\mathbf {S}_{\omega ,ii} = \sum _{k=1}^{N_v} |(\mathbf {I} - \mathbf {A}_\omega )^{-1}_{ik} |^2 ({\xi }_{\omega ,k} + {\alpha }_{\omega ,k}).
\end{eqnarray*}

Therefore, within the $\xi$–$\alpha$NET framework, spectral power emerges as a consequence of network propagation, with the Green function $(\mathbf {I} - \mathbf {A}_\omega )^{-1}$ characterizing the redistribution of power across the network. In the limit of absent inter-generator coupling ($\mathbf {A}_\omega \rightarrow 0$), the model reduces to a purely local formulation, recovering the classical vertex-wise $\xi$–$\alpha$ model applied at the source level [1,12].

#### Bayesian model inversion

We estimate the parameters of $\xi$–$\alpha$NET from rsEEG cross-spectral data in the HarMNqEEG dataset [36]. For each subject *j*, the model input is the empirical cross-spectrum $\mathbf {S}^{(j)} \in \mathbb {R}^{N_c \times N_c \times N_\omega }$, which represents the frequency-resolved sensor covariance. Parameter estimation is formulated as maximum a posteriori (MAP) estimation, combining the likelihood of the model-predicted source spectrum with priors on spectral and structural parameters (see Section S4). Closed-form analytical expressions for the score function and the Fisher information matrix are discussed in Section S5 and S20.1 of the online supplementary material, respectively.

The resulting MAP objective function is nonsmooth and nonconvex; therefore, we adopt a two-step profile-likelihood strategy. First, the structural parameters governing connectivity and delay are estimated using Bayesian optimization, constrained by physiological priors from lifespan studies [37]. This step defines subject-specific structural anchors. Second, spectral parameters are inferred using stochastic FISTA with Nesterov acceleration [38], initialized from 50 random seeds within bounded domains. Model complexity is penalized using the Bayesian information criterion, and regularization hyperparameters are tuned per subject via Bayesian optimization (see Sections S4– S7).

### Benchmarking $\xi$–$\alpha$NET: identifiability with 19 electrodes, short-term reliability and one-step superiority

**Figure 3.**
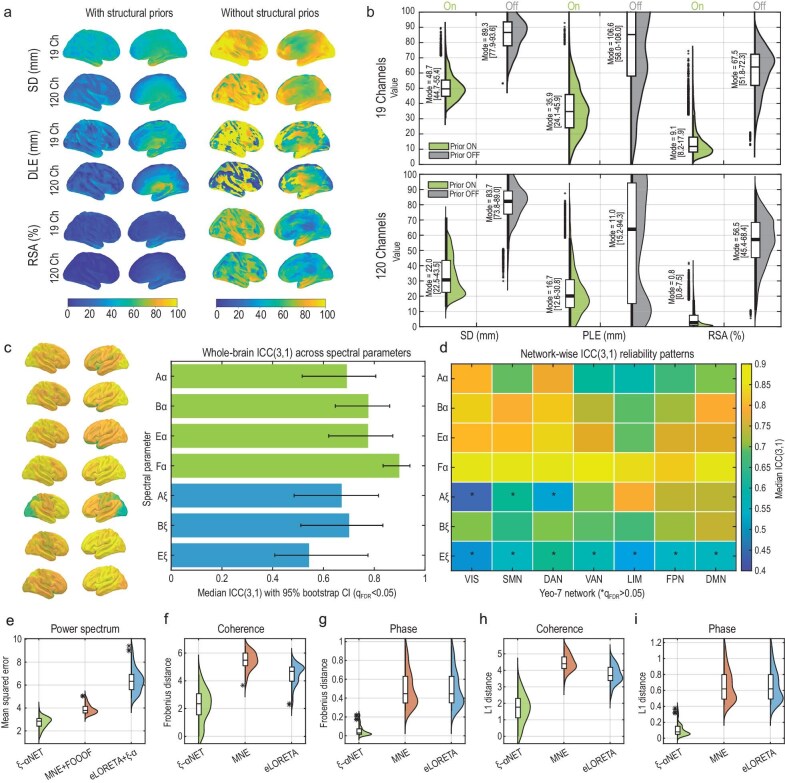
Benchmarking ${\xi }$–${\alpha }$NET. (a) Cortical maps of resolution-matrix metrics—spatial dispersion (SD), displacement/peak localization error (DLE; i.e. PLE) and ratio of spurious activity (RSA)—computed from a linearization of the model without sparse priors (upper-bound estimates of spatial error). (b) Violin plots of SD, PLE and RSA across 8003 voxels for 19- and 120-channel configurations; green = priors ON, gray = priors OFF. Priors substantially improve spatial identifiability, beyond the effect of sensor count alone. (c) Short-term test-retest reliability in an independent eyes-closed cohort ($N = 60$; two sessions 90 min apart, Ne = 19, Nv = 8003). Median intraclass correlation coefficient (ICC(3,1)) is high for $\alpha$ parameters ($\approx$0.70–0.86) and moderate for $\xi$ parameters ($\approx$0.45–0.70); among $\alpha$ features, peak alpha frequency $\mathbf {F}\alpha$ is the most reliable. (d) Network-wise ICC shows high $\alpha$ reliability across all Yeo–7NET (visual (VIS), somatomotor (SMN), dorsal attention (DAN), ventral attention/salience (VAN), limbic (LIM), frontoparietal control (FPN) and default mode (DMN)) with a gradient peaking in sensory and attention systems; $\xi$ parameters show relatively greater stability in frontal systems, with several networks not surviving FDR control. (e–i) Benchmarking of the ${\xi }$–${\alpha }$NET model in source cross-spectrum reconstruction from neural-mass simulations. Neural-mass simulations (distinct from the $\xi$–$\alpha$NET generative assumptions, Nsim = 100) show that $\xi$–$\alpha$NET outperforms two-step pipelines (MNE+FOOOF and eLORETA+$\xi$–$\alpha$) across power, coherence and phase metrics, supporting the advantage of the model’s one-step spectral inversion.

We evaluate the identifiability and stability by (i) analysing the analytical resolution matrix of a linearized $\xi$–$\alpha$NET without sparsity priors (providing an upper bound on the spatial error of the full nonlinear estimator) and (ii) performing ablation analyses that remove structural priors. The results of these simulations are show in (Fig. 3a and b), and a full description of the methods is provided in Section S9. Under these conditions, $\xi$–$\alpha$NET attains macro-regional precision with 19 electrodes: PLE = 35.9 [24.1–45.9] mm, SD = 48.7 [44.7–55.4] mm and RSA = 9.1% [8.2%–17.9%] (Fig. 3b). These values lie within, or close to, high-density EEG (hdEEG) reference ranges for methods reported by Hedrich *et al.* [39] for dSPM/MNE/sLORETA (PLE $\approx$ 25–40 mm, SD $\approx$ 35–45 mm, RSA $\approx$ 45%–55%), while exhibiting markedly lower RSA despite the lower sensor density. Using 120 electrodes further improves all metrics (PLE = 16.7 [12.6–30.8] mm, SD = 22.0 [22.5–43.5] mm, RSA = 0.8% [0.8%–7.5%]), matching or surpassing hdEEG ranges reported in [39] (Fig. 1 in Section S9). Removing the structural or delay priors degrades the performance, as shown in Fig. 3a and b ($\Delta$PLE $\approx$ +70 mm, $\Delta$SD $\approx$ 40 mm, $\Delta$RSA $\approx$ +58%), indicating that structural priors substantially enhance spatial identifiability beyond the sensor density alone.

We evaluated the impact of signal and channel noise on $\xi$–$\alpha$NET by deriving the Fisher information matrix (Section S20.1), which shows that each sensor contributes an additive negative correction proportional to its noise variance. Noisy channels therefore reduce likelihood curvature and increase posterior uncertainty under the Laplace approximation (Section S20.2). Parameter identifiability was quantified using Wald statistics derived from Laplace-approximated posteriors at the HCP-MMP1 level, corrected for multiple comparisons and summarized across Yeo-7 functional networks (Yeo-7NET) [40]. All parameters exhibited high and significant identifiability, indicating stable posterior solutions (Section S20.3).

We also assess short-term test-retest reliability using an independent open dataset ($N=60$) comprising two eyes-closed sessions recorded 90 min apart, specifically collected for reliability studies [41–43] (Fig. 3c and d). Using this dataset, we inverted the $\xi$–$\alpha$NET to estimate voxel-wise spectral parameters, which were then summarized across the Yeo-7NET. To quantify reliability, we computed the intraclass correlation coefficient ICC(3,1) [44], estimated its bootstrap confidence intervals and used a permutation test to evaluate the null hypothesis $H_0:\mathrm{ICC}\le \rho _0$ at a conservative floor $\rho _0=0.40$ (poor-to-fair reliability), followed by Benjamini–Hochberg false discovery rate (FDR) control across parameters and networks (Section S10). We observed high short-term reproducibility for $\alpha$-band parameters (median ICC $\approx 0.70$–0.86), with peak alpha frequency $\mathbf {F}\bf{\alpha }$ (PAF) being the most reliable; aperiodic $\xi$ parameters showed moderate reliability (ICC $\approx 0.45$–0.70). Network-wise, $\alpha$ spectral parameters were most reliable in sensory and attention systems (VIS, DAN, SMN), whereas $\xi$ features were relatively more stable in frontal networks, with several nonsensory networks not surviving FDR (Fig. 3c and d). These results are consistent with [42], who reported highest reliability for $\alpha$-band source reconstructions estimated with eLORETA and lower reproducibility for $\delta$-band activity, which corresponds to the $\xi$ process in our framework.

To benchmark accuracy while avoiding inverse crime [45], we used 100 neural-mass simulations whose generative assumptions differ from $\xi$–$\alpha$NET (Section S11). We then compared $\xi$–$\alpha$NET (one-step joint inversion) with two two-step pipelines: MNE$+$FOOOF [2] and eLORETA$+\xi$–$\alpha$. $\xi$–$\alpha$NET achieved lower mean-squared error for source spectra and smaller Frobenius and $L_1$ distances for coherence and phase, with tighter error distributions across runs (Fig. 3e–i). Improvements in phase recovery specifically reflect the advantage of modeling conduction delays within the inversion. These findings are consistent with the view that one-step inversion yields more accurate source estimates [17].

Together, these analyses show that $\xi$–$\alpha$NET (i) achieves spatial identifiability across 8003 cortical vertices using 19 electrodes, with further improvements at 120 electrodes, because anatomical and delay priors strongly constrain the solution; (ii) exhibits high short-term test-retest reliability, particularly for $\alpha$-band features [42]; and (iii) outperforms two-step pipelines in simulations designed to avoid model-mismatch biases (Fig. 3).

### Spectral processes exhibit distinct cortical localizations and effective connectivity patterns across the lifespan

To map the distribution of SCs and SGC, or directed connectivity patterns, of each spectral process across the Lifespan, we performed Bayesian model inversion of $\xi$–$\alpha$NET on the HarMNqEEG dataset. For each subject *j*, the observed EEG cross-spectrum $\mathbf {S}^{(j)} \in \mathbb {R}^{N_c \times N_c \times N_\omega }$ was used to estimate individualized source-level SCs via MAP optimization.

**Figure 4.**
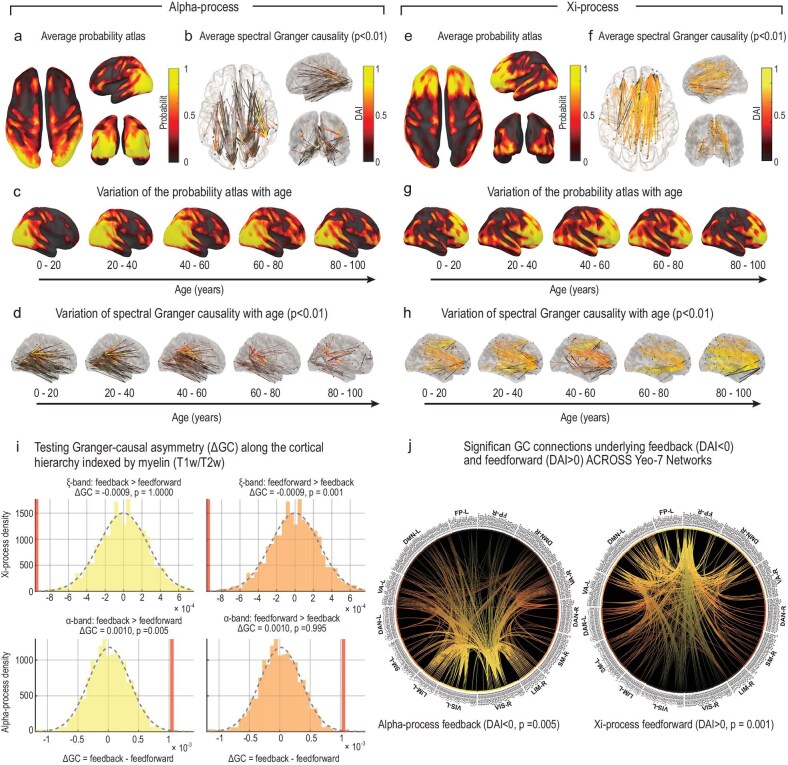
Lifespan distribution of probability atlases and spectral Granger causality for each spectral component. Effective connectivity across the lifespan, estimated with $\xi$–$\alpha$NET on the HarMNqEEG dataset ($N = 1965$), reveals opposite directional networks for the $\alpha$ and $\xi$ (aperiodic-delta) processes. (a–d) For the $\alpha$ process, the probability atlas, spectral Granger causality (directional asymmetry index, DAI) and their lifespan variation are shown. (e–h) Analogous maps for the $\xi$ process. The $\alpha$-process probability atlas localizes primarily to posterior occipito-parietal cortices, whereas the $\xi$ (aperiodic-delta) process exhibits more spatially dispersed sources, with maxima over frontal and temporal regions. These spatial configurations remain consistent across the lifespan. The DAI ($-1 =$ feedback, $+1 =$ feedforward) was computed from the spectral Granger causality and referenced to the cortical myelination hierarchy (T1w/T2w). Across 1965 participants, $\alpha$-band connectivity consistently shows feedback dominance from frontal to posterior regions, whereas $\xi$-band connectivity remains feedforward, originating in sensory regions and projecting to higher-order networks throughout the lifespan. (i) Permutation tests show that Granger-causal (GC) values are significantly greater over feedback connections for the $\alpha$ process and over feedforward connections for the $\xi$ process, given the approximate cortical myelination hierarchy ($p_{\alpha , \mathrm{Feedback}}=0.005$, $p_{\xi , \mathrm{Feedforward}}=0.001$). (j) Circular plots summarize significant directed connections within the Yeo-7NET: $\alpha$-band feedback flows from frontoparietal and attentional hubs toward sensory systems, whereas $\xi$-band feedforward ascends from sensory and limbic regions toward frontoparietal and default-mode networks, demonstrating a clear double dissociation between feedback ($\alpha$) and feedforward ($\xi$) signaling across the lifespan.

The localization probability of each SC over the cortex (probability atlas) was estimated for each frequency bin and SC using the Nadaraya–Watson kernel density estimator applied to the amplitudes $\lbrace \mathbf {A}\bf{\xi }^{(j)}, \mathbf {A}\bf{\alpha }^{(j)}\rbrace _j$ across all subjects (Section S12). As shown in Fig. 4a, c, e and g, the $\alpha$-process probability atlas across the lifespan predominantly localizes to the posterior occipito-parietal region of the cortex. By contrast, the $\xi$ (aperiodic-delta) process exhibits more spatially dispersed maxima over frontal and temporal cortices. To quantify directed interactions, we estimated frequency-resolved effective connectivity for both the $\alpha$ and $\xi$ processes using the $\xi$–$\alpha$NET spectral factorization (Equations 6–7) and the formulation of Dhamala *et al.* [35] to compute pairwise spectral Geweke–Granger causality, $\mathbf {G}_{\omega ,j\rightarrow i}$. Vertex-wise causality values were summarized within the HCP-MMP1 parcellation [24], evaluating $\alpha$ at each subject’s individual peak within the $\alpha$ band and $\xi$ at its peak in the $\delta$ range. Group-level SGC matrices were then obtained by averaging within age bins and across the full cohort (Fig. 4b, d, f and h). Statistical significance of directed connections was assessed through 5000 joint permutations of the group-mean matrix under a spatial null model ($\alpha =0.01$). Feedforward and feedback directions were defined according to the approximate cortical hierarchy gradient derived from myelination (T1w/T2w) values, as described by Burt *et al.* [46]. For each spectral process and age bin, we computed the directional asymmetry index (DAI) following Bastos *et al.* [47], weighting its sign by the cortical myelination gradient such that positive values represent feedforward and negative values represent feedback connectivity (see Section S13).

Permutation tests of Granger-causal asymmetry revealed that $\alpha$-process connectivity is significantly stronger over feedback pathways ($p_{\alpha ,\mathrm{Feedback}}=0.005$), whereas $\xi$-process connectivity is stronger over feedforward pathways ($p_{\xi ,\mathrm{Feedforward}}=0.001$) (Fig. 4i). Finally, circular graphs summarizing significant directed connections within the Yeo-7NET [40] (Fig. 4j) were constructed from the DAI-filtered adjacency matrices, displaying only those connections whose directionality (feedforward for $\xi$, feedback for $\alpha$) was significant in the permutation test. Networks are arranged from sensory (bottom) to frontal (top) regions, approximately following the cortical myelination hierarchy (Section S13).

In the left panel of Fig. 4j, the $\alpha$-process shows feedback-dominated connectivity, with edges converging from higher-order frontoparietal (FP), dorsal attention (DAN) and default-mode (DMN) networks toward visual (VIS) and somatomotor (SMN) networks. This top-down pattern reflects inhibitory and attentional control from executive to sensory systems, with the strongest feedback along the FP $\leftrightarrow$ DAN/DMN and DAN $\leftrightarrow$ VIS pathways. Conversely, in the right panel, the $\xi$ (aperiodic-delta) process exhibits feedforward-dominated connectivity, originating in sensory and limbic (LIM) regions and projecting toward FP and DMN networks. Prominent VIS $\rightarrow$ DAN/FP and SMN/LIM $\rightarrow$ DMN links indicate ascending broadband drive from perceptual and subcortical regions to higher-order systems. Together, these patterns reveal a clear double dissociation between feedback $\alpha$ and feedforward $\xi$ processes, consistent with hierarchical models of neural oscillations [47,48].

Finally, we performed a cross-dataset replication to confirm the robustness of these findings. As shown in Fig. 2 in Section S10, inversion of the model on two independent datasets—a test-retest cohort ($N = 60$, first two sessions) [41–43] and the large-scale HarMNqEEG dataset—yielded highly consistent spatial probability atlases, reflecting nearly identical cortical localization gradients ($r > 0.9$, $P_{\mathrm{spin}} < 0.01$, computed using the Neuromaps plugin in Brainstorm). Directed connectivity patterns were qualitatively preserved, with the $\alpha$ process exhibiting predominantly feedback organization and the $\xi$ process predominantly feedforward organization.

### Spectral component localization correlates with significant inverted U-shape developmental trajectories and an increase in the localization probability

$\xi$ –$\alpha$NET inversion yields, for each subject *j*, vertex-wise estimates of spectral amplitude for both the aperiodic component $\mathbf {A}\bf{\xi }^{(j)}$ and the $\alpha$-band component $\mathbf {A}\bf{\alpha }^{(j)}$, as well as the corresponding PAF map $\mathbf {F}\bf{\alpha }^{(j)}$. While age-related changes in these features have been widely reported at the sensor level [36,49–51], their spatially resolved trajectories at the cortical source level have not been systematically characterized across the lifespan.

Because the the $\xi$–$\alpha$NET generative model imposes group Lasso priors, the distributions of $\mathbf {A}\bf{\xi }^{(j)}$, $\mathbf {A}\bf{\alpha }^{(j)}$ and $\mathbf {F}\bf{\alpha }^{(j)}$ are inherently sparse, with many vertices exhibiting exact zeros. This excess of zeros in the data—known as zero inflation—violates key assumptions of conventional Gaussian-based regression. Therefore, to model the developmental trajectories of the SCs accurately, we employed a zero-inflated Gaussian (ZIG) regression model. The use of this type of regression model enables us to account for sparsity, non-negativity and nonlinear age effects, providing a robust statistical basis for mapping developmental trends in source-resolved spectral activity [52].

We employed a vertex-wise ZIG regression model of the form (Section S14)


(9) \begin{eqnarray*}
&&Y_{ij} \sim \mathrm{ZIG}( \mu _{ij}, \sigma _{ij}^2, \pi _{ij} ),\nonumber \\
&&\mu _{ij} = \beta _{0,i} + \beta _{1,i} a_j + \beta _{2,i} a_j^2,\nonumber \\
&&{\rm logit}(\pi _{ij}) = \gamma _{0,i} + \gamma _{1,i} a_j,
\end{eqnarray*}

where $Y_{ij}$ denotes the response variable—either $\mathbf {A}\bf{\xi }$, $\mathbf {A}\bf{\alpha }$ or $\mathbf {F}\bf{\alpha }$—at vertex *i* for subject *j*, and $a_j$ is the subject’s age. The function ${\rm logit}(p) = \log (p / (1-p))$ denotes the logit link, and $\mu _{ij}$ is the conditional mean (CM), which captures the expected value of the positive (nonzero) portion of the distribution.

**Figure 5.**
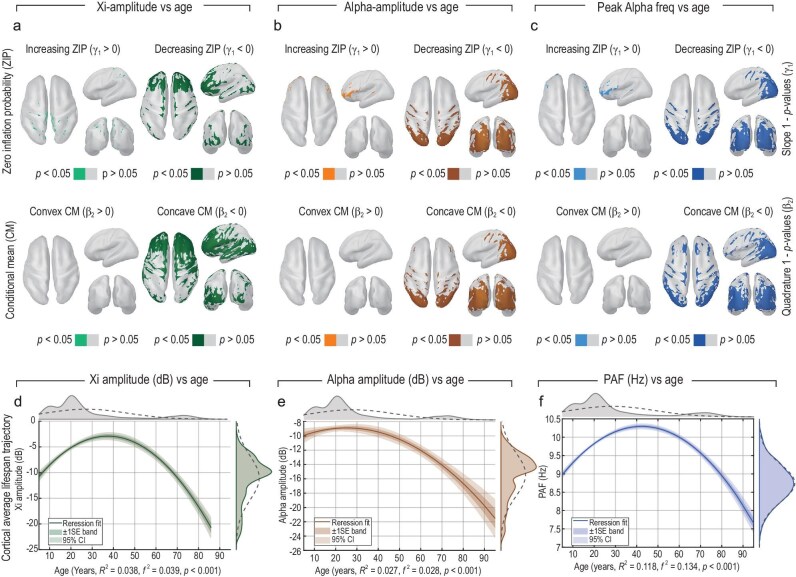
Developmental atlas of spectral EEG components estimated with zero-inflated Gaussian (ZIG) regression on HarMNqEEG ($N=1965$). All panels use vertex-wise ZIG models that jointly estimate the zero-inflation probability (ZIP) and the conditional mean (CM) as functions of age. Maps display significance masks ($p<0.05$) for two distinct effects: (i) the age slope $\gamma _{1}$ of the ZIP component (change in zero inflation with age; coloured = significant, grey = not significant), and (ii) the age quadratic curvature $\beta _{2}$ of the CM component (concavity or convexity of the nonzero mean trajectory with age). (a) The $\xi$ amplitude ($\mathbf {A}\bf{\xi }$): ZIP decreases widely with age ($\gamma _{1}<0$), indicating more frequent detectability, while the CM shows predominantly concave (inverted-U) trajectories ($\beta _{2}<0$) with a widespread cortical distribution. (b) The $\alpha$ amplitude ($\mathbf {A}\bf{\alpha }$): ZIP increases mainly in the frontal cortex and decreases in the occipital cortex, revealing an anterior-posterior gradient; CM curvature is largely concave posteriorly, consistent with growth in early to mid-adulthood followed by decline. (c) Peak $\alpha$ frequency (PAF; $\mathbf {F}\bf{\alpha }$): using the same ZIG formulation, maps show negative age slopes predominantly in posterior regions ($\gamma _{1}<0$) and concave CM curvature concentrated occipitally ($\beta _{2}<0$), consistent with lifespan slowing that is most pronounced in the posterior cortex. (d–f) Cortical-average trajectories (density plots; robust quadratic regression): $\mathbf {A}\bf{\xi }$, $\mathbf {A}\bf{\alpha }$ and $\mathbf {F}\bf{\alpha }$ exhibit statistically significant inverted-U patterns across the lifespan. Effect sizes are small for amplitudes and larger for PAF (panel labels report $R^{2}$ and $f^{2}$; all $p<0.001$). For each trajectory, both 95% confidence bands and $\pm$1 SE envelopes are shown.

Figure 5 presents the developmental atlas of spectral EEG components estimated using ZIG regression applied to the principal spectral parameters of the $\xi$–$\alpha$NET model across the full HarMNqEEG dataset. Each vertex-wise ZIG model jointly estimates the ZIP and the CM as smooth functions of age, thereby disentangling effects related to the detectability of spectral components from those governing their nonzero amplitudes.

Panels (a)–(c) of Fig. 5 display significance maps ($p<0.05$) for two age effects: the ZIP slope ($\gamma _{1}$; top row), indicating how the probability of zero inflation changes with age, and the quadratic curvature of the CM ($\beta _{2}$; bottom row), reflecting whether the nonzero mean follows a convex or concave (inverted-U) trajectory.

For the $\mathbf {A}\bf{\xi }$ amplitude (Fig. 5a), ZIP decreases widely with age ($\gamma _{1} < 0$), indicating that aperiodic $\xi$ activity becomes increasingly detectable across the cortex in older individuals. The CM component exhibits predominantly concave trajectories ($\beta _{2} < 0$), indicating that $\mathbf {A}\bf{\xi }$ follows a trajectory of growth and decline. The $\mathbf {A}\bf{\alpha }$ amplitude (Fig. 5b) reveals a pronounced anterior-posterior gradient: ZIP increases in frontal regions but decreases occipitally, suggesting reduced detectability of $\alpha$ rhythms in anterior cortex with age. The CM curvature remains concave in posterior regions ($\beta _{2} < 0$), consistent with growth through early to mid-adulthood followed by decline in regions generating $\alpha$ oscillations. For peak $\alpha$ frequency ($\mathbf {F}\bf{\alpha }$; PAF; Fig. 5c), ZIP slopes are predominantly negative in the posterior cortex ($\gamma _{1} < 0$), while CM curvature is concave and confined to occipital regions ($\beta _{2} < 0$), consistent with a lifespan slowing of PAF that is most pronounced in posterior regions. Finally, panels (d)–(f) of Fig. 5 summarize cortical-average lifespan trajectories using robust quadratic regression. All three spectral variables—$\mathbf {A}\bf{\xi }$, $\mathbf {A}\bf{\alpha }$ and $\mathbf {F}\bf{\alpha }$—exhibit statistically significant inverted-U patterns (all $p<0.001$), with small effect sizes for amplitudes and larger effect sizes for PAF.

Together, these findings demonstrate that both aperiodic and $\alpha$ EEG components follow nonmonotonic developmental trajectories that vary spatially across the cortex. The results replicate and extend canonical sensor-level observations of PAF slowing across the lifespan to the source level, revealing that posterior-anterior gradients in ZIP modulate where $\alpha$ activity remains detectable. Overall, the localization of spectral components correlates with significant inverted-U developmental trajectories.

### Estimated conduction delays are negatively correlated with the peak alpha frequency and with independently reported cortical myelin (T1w/T2w) data

Conduction delays are governed by axonal diameter and myelin thickness, which jointly determine the velocity of action-potential propagation along white-matter tracts. Myelination—the ensheathing of axons by lipid-rich membranes—enhances conduction speed and exhibits a characteristic nonmonotonic trajectory across the lifespan: it increases from childhood through early adulthood, plateaus in midlife and declines with aging, yielding an inverted-U pattern of cortical myelin content [53,54]. This trajectory, consistently observed in T1w/T2w MRI studies, modulates conduction velocity and the temporal synchronization of neural activity. In line with the Rushton model [55], myelin thickness scales inversely with the square of the mean conduction delay ($\propto 1/\langle \tau \rangle ^2$). We therefore defined an EEG-based *myelination proxy* as ${1}/{\langle \tau \rangle ^2}$, expected to reproduce the same inverted-U lifespan trajectory.

**Figure 6.**
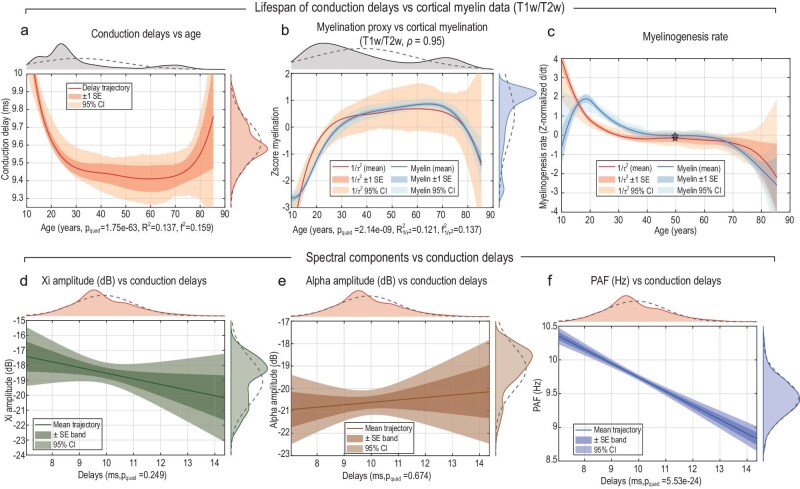
Lifespan dynamics of conduction delays, cortical myelination and spectral components. Lifespan trajectories were estimated using eight piecewise cubic B-spline basis functions, with smoothing parameters selected via Akaike information criterion (AIC) minimization. (a) Mean conduction delays estimated by $\xi$–$\alpha$NET across the HarMNqEEG dataset ($N = 1965$) show a U-shaped trajectory with age ($p_{\mathrm{quad}} = 1.75\times 10^{-63}$, estimated by fitting a robust quadrtic model). (b) *Z*-scored myelination proxy ($1/\mathrm{Delay}^2$, orange) and empirical cortical myelination (T1w/T2w, blue) from Grydeland *et al.* [53] ($N = 484$) exhibit highly correlated inverted-U trajectories ($\rho = 0.95$), with $R^2_{\mathrm{myelin}} = 0.611$ and $f^2_{\mathrm{myelin}} = 1.570$. (c) First derivatives of spline fits (myelinogenesis rates) converge near 50 years of age, marking midlife stabilization of cortical myelination. (d–f) Associations between conduction delays and spectral parameters: only $\mathbf {F}\bf{\alpha }$ (PAF) declines significantly with increasing delays ($p = 5.53\times 10^{-24}$).

To test this electrophysiological prediction, we estimated conduction delays in 1965 participants from the HarMNqEEG dataset using the $\xi$–$\alpha$NET model. For each participant *j*, the mean conduction delay $\langle \tau \rangle ^{(j)}$ was obtained by averaging the estimated delay matrix across all neurotracts ($\langle \tau \rangle ^{(j)} = \sum _{i}\sum _k \mathbf {D}_{ik}^{(j)}$), and the corresponding myelination proxy was defined as ${1}/{(\langle \tau \rangle ^{(j)})^2}$ (see Section S15). Lifespan trajectories were modeled as functions of age $a_j$ using eight piecewise cubic B-spline basis functions, with optimal smoothing selected via AIC, following Grydeland *et al.* [53]. The significance of quadratic curvature was assessed using robust quadratic regression. The resulting trajectory revealed a pronounced U-shaped pattern of conduction delays across the lifespan ($p_{\mathrm{quad}} = 1.75\times 10^{-63}$), marked by rapid acceleration in early development, stabilization in midlife and slowing in older age (Fig. 6a).

To compare the electrophysiological myelination proxy with independent MRI-derived measures, we used the cortical T1w/T2w myelination dataset from Grydeland *et al.* [53] ($N = 484$), also reported in Fig. 4a of de Faria *et al.* [54]. Both the EEG-based proxy and the MRI-derived myelin measure were *z*-score normalized prior to modeling. The EEG-derived proxy exhibited a significant inverted-U lifespan trajectory ($p_{\mathrm{quad}} = 2.14\times 10^{-9}$), while the MRI curve exhibited $R^2_{\mathrm{myelin}} = 0.611$ and $f^2_{\mathrm{myelin}} = 1.57$. The correlation between the two spline trajectories was high ($\rho = 0.95$), indicating strong convergence of their developmental patterns (Fig. 6b). However, the EEG-derived proxy displayed wider 95% confidence intervals and a smaller effect size ($R^2 = 0.121$, $f^2 = 0.137$), reflecting the greater noise inherent in electrophysiological estimates compared with MRI-based myelin measurements.

The first derivatives of the spline fits—reflecting myelinogenesis rates—for both cortical myelination and the electrophysiological myelination proxy followed qualitatively similar trajectories for ages above 15 years. In both cases, the rate approached zero around 50 years of age, indicating the midlife stabilization of cortical myelination consistently reported in MRI studies and mirrored by our proxy (Fig. 6c). Minor discrepancies at younger ages likely arise from spline boundary effects, the nonlinear relationship between true myelin content and its proxy ($\mathrm{Myelin} \sim A(1 - \exp (-B/\tau ^2))$, where $1/\tau ^2$ is a first-order Taylor approximation) and the greater noise inherent in EEG-derived conduction delays compared with MRI-based measures.

Regression analyses further revealed a significant negative relationship between conduction delays and peak alpha frequency ($\mathbf {F}\bf{\alpha }$, $p = 5.53 \times 10^ {-24}$). In contrast, neither $\mathbf {A}\bf{\xi }$ ($p = 0.25$) nor $\mathbf {A}\bf{\alpha }$ ($p = 0.67$) showed significant associations (Fig. 6d–f). Faster conduction—reflecting higher myelin content—was thus associated with higher $\mathbf {F}\bf{\alpha }$ values, supporting the view that alpha-rhythm frequency depends on axonal conduction efficiency. This finding aligns with earlier observations that diffusion-tensor-derived fractional anisotropy correlates with PAF in the Cuban Human Brain Mapping Project [56], as well as with theoretical predictions from thalamocortical modeling [20,21,57], in which conduction delays govern the emergence of oscillatory alpha modes.

Together, these results demonstrate that $\xi$–$\alpha$NET can infer physiologically meaningful conduction delays from resting-state EEG. The inferred trajectories parallel those of cortical myelination measured by MRI and reveal a tight structural-functional coupling, whereby white-matter integrity constrains the spectral dynamics of cortical rhythms. The strong inverse relationship between $\mathbf {F}\bf{\alpha }$ and conduction delay highlights PAF as a sensitive functional marker of myelin-dependent transmission speed across the human lifespan.

## CONCLUSION AND DISCUSSION

We introduce $\xi$–$\alpha$NET, a generative model of EEG cortical activity that represents each spectral component as a sparse, structurally constrained network of independent Hida–Matérn processes in the time domain. By integrating the sparse connectome derived from dMRI [58] and conduction delays estimated from CCEP [37], the model captures the spatiotemporal dependencies among cortical generators and yields Lorentzian spectral profiles describing spectral processes that reduce, in the absence of priors, to the traditional SCM framework of Pascual-Marqui *et al.* [1,14,56,59]. This framework unifies spectral decomposition and effective connectivity mapping while explicitly incorporating anatomical and delay priors in the generative model—an aspect neglected in existing SCM approaches [2,11,14]. Analyses demonstrate that $\xi$–$\alpha$NET achieves good spatial resolution, high short-term test-retest reliability—particularly for $\alpha$-rhythm features [42]—and superior accuracy relative to standard two-step pipelines in simulations controlling for model-mismatch biases (Fig. 3).

Using Bayesian inversion, $\xi$–$\alpha$NET revealed distinct lifespan trajectories and directional architectures for the $\xi$ (aperiodic-delta) and $\alpha$ processes across the HarMNqEEG. The $\xi$ component exhibited widespread cortical engagement and a predominant feedforward organization, under the cortical myelination hierarchy of Burt *et al.* [46], with ascending influences from sensory to higher-order regions. In contrast, the $\alpha$ rhythm localized to posterior cortices and exhibited a feedback organization across development, consistent with the pattern reported by Michalareas *et al.* [48] (Fig. 4). Both $\alpha$ amplitude and PAF followed inverted U-shaped age trajectories, reflecting maturation and subsequent slowing of cortical feedback loops, in line with previous empirical and theoretical findings (Fig. 5). Conduction-delay estimates were negatively correlated with PAF, consistent with earlier empirical and theoretical work [20,56,57].

In addition, conduction delays closely mirrored empirical cortical myelination (T1w/T2w) reported by Grydeland *et al.* [53], exhibiting highly correlated inverted-U trajectories across the lifespan. The first derivatives of these spline fits—representing myelinogenesis rates—showed similar age-dependent patterns, approaching zero around midlife and marking the stabilization of cortical myelination observed in MRI studies and reflected in our electrophysiological proxy (Fig. 6).

We believe that a unified generative framework for spectral component modeling is necessary to explain how distinct spectral processes arise within realistic brain networks and how they give rise to effective connectivity patterns grounded in anatomy across the lifespan. By jointly estimating structural and spectral parameters while incorporating structural priors, this approach helps bridge the gap between structure, dynamics and function. In doing so, it moves beyond purely descriptive analyses and provides a pathway for using EEG to investigate how processes such as myelination regulate spectral dynamics. We expect that individually estimated $\xi$–$\alpha$NET parameters may serve as biomarkers for neurological conditions. As illustrated by the Parkinson’s disease analysis in Section S21, subject-level estimates reveal disease-related alterations in spectral dynamics in a concrete clinical setting, specifically demonstrating a slowing of the alpha rhythm in patients with Parkinson’s disease relative to age-matched controls from HarMNqEEG. Building on this proof of concept, future work will focus on constructing normative reference datasets to enable systematic detection, quantification and interpretation of patient-specific deviations from the norm within a clinically meaningful framework.

The $\xi$–$\alpha$NET framework has several limitations. Its reduction of thalamocortical dynamics and cortico-cortical interactions to a linear MVAR model with colored Hida–Matérn innovations cannot capture the nonlinear excitatory-inhibitory interactions, attractor dynamics and state-dependent transitions that characterize real cortical activity. Incorporating nonlinear activation functions could improve biological realism. The use of a Dirac delta function to represent conduction delays also oversimplifies temporal dispersion, which may be better modeled using broader delay distributions, such as those from the exponential family [25]. Furthermore, our analyses relied on low-density EEG, which limits spatial resolution compared with high-density recordings or MEG. Although the model can, in principle, be extended to MEG by modifying the lead field, this has not yet been tested. The present work also focuses exclusively on two spectral components—the aperiodic ($\xi$) and $\alpha$ processes—without assessing the role or directionality of higher-frequency rhythms, such as gamma, which may contribute critically to feedforward signaling. Finally, the current formulation assumes stationarity, which restricts its applicability to transient or task-related dynamics [9]. Addressing these limitations—including nonlinear modeling, richer delay priors, higher-density data and extended spectral coverage—will be essential to further enhance $\xi$–$\alpha$NET’s generality and biological fidelity.

## MATERIALS AND METHODS

### Resources and code

All analyses were performed on a high-performance server (52 CPUs, 256 GB RAM). The full $\xi$–$\alpha$NET pipeline—including parameter estimation, visualization, regression and documentation—is available on GitHub (Xi-AlphaNET). Reproducibility resources (commit and tag, MATLAB environment file and minimal scripts for every figure and statistical analysis) are detailed in Section S19.

### Data

Both structural and electrophysiological data informed the model. Structural priors were derived from the anatomical connectivity matrix $\bar{\mathbf {C}}$ of Rosen *et al.* [58] and available via Zenodo, and from the axonal conduction-delay matrix $\bar{\mathbf {D}}$ from the F-TRACT consortium, estimated using CCEP [37]. The head model, source model and lead-field matrix were obtained using the Ciftistorm-Brainstorm pipeline implemented on the *fsaverage* cortical surface ($N_v = 8003$ vertices) [60]. Empirical data were drawn from the HarMNqEEG dataset [36], which provides resting-state EEG cross-spectral tensors ($N_c = 19$, $N_\omega = 47$) from 1965 participants aged 5–100 years across nine countries, as well as from the short-term test-retest dataset [41–43]. Preprocessing followed the procedures described by Li *et al.* [36], including average referencing, removal of global scaling and cross-spectral regularization (Sections S17– S18). Cortical myelination (T1w/T2w) values were obtained from the Human Connectome Project (S1200 release) and summarized over the HCP-MMP1 parcellation using Neuromaps. In addition, the raw cortical myelination dataset ($N = 484$) from Grydeland *et al.* [53] was used to estimate myelination and myelinogenesis trajectories and compared with our EEG-based myelination proxy.

All $\xi$–$\alpha$NET-derived spectral and structural parameters from HarMNqEEG are publicly available (Xi-AlphaNET Data, $\sim$60 GB). The repository includes source power spectra, cross-spectral matrices, cortical activation and connectivity maps, conduction delay matrices and all structural priors necessary to reproduce the results for both the HarMNqEEG [36] and test-retest datasets [42,61]. A graphical interface for visualization is provided within the Xi-AlphaNET app.

## Supplementary Material

nwag076_Supplemental_File
