# Supplementary material for: Lifespan development of EEG alpha and aperiodic component sources is shaped by the connectome and axonal delays
Source: Natl Sci Rev. 2026 Feb 6;13(7):nwag076. doi: 10.1093/nsr/nwag076 (PMC13105192; doi:10.1093/nsr/nwag076)
Supplement: nwag076_Supplemental_File [file nwag076_supplemental_file.pdf]

Supplementary Materials: Lifespan Development of EEG Alpha and Aperiodic Component Sources is Shaped by the Connectome and Axonal Delays

Ronaldo Garcia Reyes[1,2,5,*,†], Ariosky Areaces Gonzales[1,4,5], Ying Wang[1,5], Yu Jin[1,5], Shahwar Yasir [1,5], Maria Luisa Bringas-Vega[1,5], Mitchell Valdes-Sosa[2,5], Cheng Luo[1,5], Peng Xu[6], Viktor Jirsa[7], Dezhong Yao[1,5], Ludovico Minati[1,3,5], Pedro A. Valdes–Sosa[1,2,5,*,†]

[1] Clinical Hospital of Chengdu Brain Science Institute, University of Electronic Science and Technology of China, 610054, Chengdu, China; [2] Neuroinformatics, Cuban Neurosciences Center, 11300, Havana, Cuba; [3] Center for Mind/Brain Science (CIMeC), University of Trento, 38123, Trento, Italy; [4] University "Hermanos Saiz Montes de Oca" of Pinar del Río, Pinar del Río, Cuba; [5] China-Cuba Belt and Road Joint Laboratory on Neurotechnology and Brain-Apparatus Communication, University of Electronic Science and Technology of China, Chengdu, P. R. China; [6] Laboratory for Brain Science and Artificial Intelligence, Southwest University of Science and Technology, Mianyang 621010, China; [7] Aix Marseille Université, Institut National de la Santé et de la Recherche Médicale, Institut de Neurosciences des Systèmes (INS) UMR1106; Marseille 13005, France.

Contents

1 Notation 4

2 ξ-αNET generative model in time Domain 5

3 Spectral Densities of the Voxel-Wise Hida-Matérn Processes 6

4 Derivation of the Frequency-Domain MAP Estimator for ξ-αNET 7

5 Gradient Derivation (Score Function): $\partial_{\sigma^2_\omega} f$ and $\partial_{a_{ij}} f$ 9

6 ξ-αNET Spectral Components Search Space 11

7 Selection of the Regularization Space 11

8 Automatic selection of data-driven thresholds by Expectation Maximisation 12

9 Resolution–matrix derivation & Ablation setup 14

10 Test-retest reliability and cross-dataset replication 14

11 Delayed Neural Mass Model with Structural and Delay Priors 15

12 Nonparametric estimation of the Probability Atlas 16

13 Estimation of Spectral granger causality 16

14 Voxel-wise Zero-Inflated Gaussian (ZIG) Model and Estimation Procedure 17

15 Estimation of Lifespan Trajectories of Conduction Delays 18

16 Gaussian-Process Estimation of ROI-Level Conduction Delays and Projection to Voxel Space 19

17 Average Reference Transformation 20

18 Global-Scale Factor (GSF) Correction 20

19 Code and Data Reproducibility 21

19.1 Data Availability 21

19.2 How to reproduce the analysis of each figure of the paper 21

20 Laplace-Based Posterior Inference and Information-Geometric Identifiability 22

20.1 Laplace Approximation of Posterior Distribution & Fisher Information Matrix 22

20.2 Impact of Signal and Channel Quality on the Xi-AlphaNET estimation 22

20.3 Posterior Uncertainty and Parameter Identifiability 23

21 Individual-Level ξ–αNET Inference and Clinical Translation: A Parkinson's Disease Case Study **24**

1 Notation

Table 1: Notation for the ξ–αNET Model: Variables, Spectral Parameters, Covariance Kernels, and Operators

Symbol / Operator	Dimension	Description
**Model Variables and Parameters**		
$\mathbf{v}_t$	$\mathbb{R}^{N_c \times 1}$	EEG signal at sensors (time domain)
$\mathbf{K}$	$\mathbb{R}^{N_c \times N_v}$	Leadfield matrix mapping sources to sensors
$\mathbf{j}_t$, $\mathbf{j}_t^\xi$, $\mathbf{j}_t^\alpha$	$\mathbb{R}^{N_v \times 1}$	Source activity: total, aperiodic, and alpha (time domain)
$\mathbf{n}_t^v$	$\mathbb{R}^{N_c \times 1}$	Sensor noise (time domain)
$\mathbf{u}_t^\xi$, $\mathbf{u}_t^\alpha$	$\mathbb{R}^{N_v \times 1}$	Innovations driving aperiodic and alpha components
w_C, w_D	Scalar	Global scaling factors for connectivity and delays
$\bar{\mathbf{C}}$	$\mathbb{R}^{N_v \times N_v}$	Baseline anatomical connectivity matrix
$\bar{\mathbf{D}}$	$\mathbb{R}^{N_v \times N_v}$	Baseline conduction delay matrix
**Frequency-Domain Quantities**		
$\mathbf{v}_\omega$	$\mathbb{C}^{N_c \times 1}$	EEG signal at sensors (frequency domain)
$\mathbf{j}_\omega$, $\mathbf{j}_\omega^\xi$, $\mathbf{j}_\omega^\alpha$	$\mathbb{C}^{N_v \times 1}$	Source activity in frequency domain
$\mathbf{u}_\omega^\xi$	$\mathbb{C}^{N_v \times 1}$	Innovation term (aperiodic) in frequency domain
$\mathbf{u}_\omega^\alpha$	$\mathbb{C}^{N_v \times 1}$	Innovation term (alpha) in frequency domain
$\mathbf{u}_\omega^\xi \sim \mathcal{CN}(\mathbf{0}, \mathrm{diag}(\boldsymbol{\xi}_\omega))$	—	Distribution of aperiodic innovation in frequency domain
$\mathbf{u}_\omega^\alpha \sim \mathcal{CN}(\mathbf{0}, \mathrm{diag}(\boldsymbol{\alpha}_\omega))$	—	Distribution of alpha innovation in frequency domain
$\mathbf{n}_\omega^v$	$\mathbb{C}^{N_c \times 1}$	Sensor noise in frequency domain
$\mathbf{n}_\omega^v \sim \mathcal{CN}(\mathbf{0}, \sigma_\omega^2 \mathbf{I}_{N_c})$	—	Distribution of sensor noise in frequency domain
$\mathbf{A}_\omega$	$\mathbb{C}^{N_v \times N_v}$	Frequency-domain connectivity operator
$\mathbf{T}_\omega$	$\mathbb{C}^{N_c \times N_v}$	Transfer operator mapping source spectra to sensors
$\boldsymbol{\xi}_\omega$, $\boldsymbol{\alpha}_\omega$	$\mathbb{R}^{N_v \times 1}$	Parametric source spectral densities
$\psi(\omega \mid A, B, E, F)$	—	Generalized Lorentzian spectrum defined by amplitude A, bandwidth B, exponent E, and frequency shift F
**Spectral Parameters and Their Vector Forms**		
$A\xi_i$, $A\alpha_i$	Scalar	Amplitudes (in dB)
$B\xi_i$, $B\alpha_i$	Scalar	Spectral bandwidths (sec^2)
$E\xi_i$, $E\alpha_i$	Scalar	Spectral decay exponents
$F\alpha_i$	Scalar	Peak Alpha Frequency (Hz)
$\mathbf{A}\boldsymbol{\xi}$, $\mathbf{A}\boldsymbol{\alpha}$	$\mathbb{R}^{N_v \times 1}$	Amplitude vectors across voxels
$\mathbf{B}\boldsymbol{\xi}$, $\mathbf{B}\boldsymbol{\alpha}$	$\mathbb{R}^{N_v \times 1}$	Bandwidth vectors across voxels
$\mathbf{E}\boldsymbol{\xi}$, $\mathbf{E}\boldsymbol{\alpha}$	$\mathbb{R}^{N_v \times 1}$	Spectral decay exponent vectors
$\mathbf{F}\boldsymbol{\alpha}$	$\mathbb{R}^{N_v \times 1}$	Vector of Peak Alpha Frequencies
$\mathbf{e}$	$\mathbb{R}^{N_v \times 3}$	Concatenated vector: $\{\mathbf{A}\boldsymbol{\xi}, \mathbf{B}\boldsymbol{\xi}, \mathbf{E}\boldsymbol{\xi}\}$
$\mathbf{a}$	$\mathbb{R}^{N_v \times 4}$	Concatenated vector: $\{\mathbf{A}\boldsymbol{\alpha}, \mathbf{B}\boldsymbol{\alpha}, \mathbf{E}\boldsymbol{\alpha}, \mathbf{F}\boldsymbol{\alpha}\}$
**Covariance Kernels and Processes**		
$\mathbf{HM}^\eta(\tau)$	—	Hida-Matérn covariance kernel for component η
$M^\eta(\tau)$	—	Standard Matérn covariance kernel (non-cosine modulated)
$\mathbf{u}_t^\eta$	—	Gaussian process driven by $\mathbf{HM}^\eta(\tau)$
$\mathcal{GP}(\mathbf{0}, \mathbf{HM}^\eta(\tau))$	—	Gaussian process with Hida-Matérn covariance kernel

Table 2: Mathematical Operators and Conventions

Symbol / Operator	Dimension	Description
**Operators and Conventions**		
$\odot$	—	Hadamard (element-wise) product
$\delta(\cdot)$	—	Dirac delta function
$\mathcal{F}^{-1}$	—	Inverse Fourier Transform
$\dagger$	—	Hermitian (conjugate transpose)
$\mathbf{I}_n$	$\mathbb{R}^{n\times n}$	Identity matrix of size n
$\mathrm{diag}(\cdot)$	—	Operator that maps a vector to a diagonal matrix
$\mathrm{vec}_r(\cdot)$	—	Row-wise vectorization of a matrix
$\mathrm{tr}(\cdot)$	—	Trace of a matrix (sum of diagonal elements)
$\log\lvert\cdot\rvert$	—	Log-determinant of a matrix
$\lvert\cdot\rvert$	—	Matrix determinant
$\exp(\cdot)$	—	Matrix or element-wise exponential
$\lVert\cdot\rVert_2$	—	Euclidean (L2) norm
$\lVert\cdot\rVert_{2,1}$	—	Group Lasso norm (sum of row-wise norms)
$\circledast$	—	Convolution operator in the time domain

Table 3: Optimization and Differential Notation in the ξ–αNET Model

Symbol / Operator	Dimension	Description
$d_x f$	Same as f	Differential of function f with respect to x
$\partial_x f$	Same as f	Partial derivative of f with respect to x
$\nabla_x f$	Same as x	Gradient of f with respect to variable x
$\nabla_{\boldsymbol{\sigma}^2} f$	$\mathbb{R}^{N_\omega\times 1}$	Gradient w.r.t. noise spectrum
$\nabla_{\mathbf{e}} f$	$\mathbb{R}^{N_v\times 3}$	Gradient w.r.t. ξ spectral parameters
$\nabla_{\mathbf{a}} f$	$\mathbb{R}^{N_v\times 4}$	Gradient w.r.t. α spectral parameters
$d\log\lvert\mathbf{A}\rvert$	—	Matrix identity: $\mathrm{tr}(\mathbf{A}^{-1}d\mathbf{A})$
$d\mathbf{A}^{-1}$	—	Matrix inverse differential: $-\mathbf{A}^{-1}(d\mathbf{A})\mathbf{A}^{-1}$
$\mathrm{prox}_{\lambda g}(v)$	Same as v	Proximal operator for penalty g at v
$\left(1-\frac{\lambda}{\lVert v\rVert}\right)_+ v$	Same as v	Group Lasso block soft-thresholding rule

2 ξ-αNET generative model in time Domain

Using matrix notation, the full generative model of ξ-αNET its:

$$\begin{aligned}
&\mathbf{v}_t = \mathbf{K}\mathbf{j}_t + \mathbf{n}_t^v, \quad \mathbf{j}_t = \mathbf{j}_t^\xi + \mathbf{j}_t^\alpha,\\
&\mathbf{j}_t^\xi = (\mathbf{A}\circledast\mathbf{j}^\xi)_t + \mathbf{u}_t^\xi, \quad \mathbf{j}_t^\alpha = (\mathbf{A}\circledast\mathbf{j}^\alpha)_t + \mathbf{u}_t^\alpha,\\
&\mathbf{A}_\tau = \mathbf{C}\odot\delta(\tau-\mathbf{D}), \quad \mathbf{C} = w_C\bar{\mathbf{C}}, \quad \mathbf{D} = w_D\bar{\mathbf{D}},\\
&\mathbf{n}_t^v \sim \mathcal{GP}(\mathbf{0}, \sigma_\tau^2\mathbf{I}_{N_c}\delta(\tau)),\\
&\mathbf{u}_t^\xi \sim \mathcal{GP}(\mathbf{0}, \mathbf{HM}^\xi(\tau)), \quad \mathbf{u}_t^\alpha \sim \mathcal{GP}(\mathbf{0}, \mathbf{HM}^\alpha(\tau)),\\
&\mathbf{e} \sim \exp(\lVert\mathbf{e}\rVert_{2,1} \mid \lambda_2), \quad \mathbf{a} \sim \exp(\lVert\mathbf{a}\rVert_{2,1} \mid \lambda_3), \quad \boldsymbol{\sigma} \sim \exp(\lVert\boldsymbol{\sigma}\rVert_2^2 \mid \lambda_1).
\end{aligned}$$

Where $\mathbf{v}_t \in \mathbb{R}^{N_c}$ denotes the EEG sensor measurements at time t. The cortical source activity $\mathbf{j}_t \in \mathbb{R}^{N_v}$ is decomposed into an aperiodic component (ξ) and an oscillatory component (α). The lead-field matrix $\mathbf{K} \in \mathbb{R}^{N_c\times N_v}$ maps cortical sources to sensors. The autoregressive coefficients $\mathbf{A}_\tau$ are defined element-wise through the Dirac delta function and constructed using voxelwise anatomical connectivity $\mathbf{C}$ and conduction delay matrix $\mathbf{D}$. These matrices are defined as modulated forms of the baseline structural connectivity $\bar{\mathbf{C}}$ and baseline conduction delays $\bar{\mathbf{D}}$:

$$\mathbf{C} = w_C\,\bar{\mathbf{C}}, \quad \mathbf{D} = w_D\,\bar{\mathbf{D}}.$$

Here, w_C and w_D are scalar modulation parameters that allow data-driven tuning of anatomical connectivity strength and conduction delays. The product $\odot$ denotes the Hadamard (element-wise) product, and $\circledast$ denotes temporal convolution. The sensor noise $\mathbf{n}_t^v$ is modeled as a zero-mean Gaussian process:

$$\mathbf{n}_t^v \sim \mathcal{GP}\Big(\mathbf{0}, \sigma_\tau^2 \mathbf{I}_{N_c}\, \delta(\tau)\Big),$$

where σ_τ^2 represents the variance of the noise process per sensor, and $\mathbf{I}_{N_c} \in \mathbb{R}^{N_c \times N_c}$ ensures that the noise process is temporally white and spatially uncorrelated. The voxelwise stochastic innovations $\mathbf{u}_t^\xi$ and $\mathbf{u}_t^\alpha$ are modeled as zero-mean Gaussian processes governed by Hida-Matérn covariance functions:

$$\mathbf{u}_t^\xi \sim \mathcal{GP}\Big(\mathbf{0}, \mathbf{HM}^\xi(\tau)\Big), \quad \mathbf{u}_t^\alpha \sim \mathcal{GP}\Big(\mathbf{0}, \mathbf{HM}^\alpha(\tau)\Big).$$

For the aperiodic component:

$$\mathbf{HM}_{ik}^\xi(\tau) = \delta_{i,k}\, \frac{2\sqrt{\pi} e_{1,i}}{\sqrt{e_{2,i}}\,\Gamma(e_{3,i})} \left(\frac{\pi|\tau|}{\sqrt{e_{2,i}}}\right)^{e_{3,i}-\frac{1}{2}} K_{e_{3,i}-\frac{1}{2}}\Big(\frac{2\pi|\tau|}{\sqrt{e_{2,i}}}\Big).$$

For the alpha component:

$$\mathbf{HM}_{ik}^\alpha(\tau) = \delta_{i,k}\, \cos(2\pi a_{4,i}\tau)\, \frac{2\sqrt{\pi} a_{1,i}}{\sqrt{a_{2,i}}\,\Gamma(a_{3,i})} \left(\frac{\pi|\tau|}{\sqrt{a_{2,i}}}\right)^{a_{3,i}-\frac{1}{2}} K_{a_{3,i}-\frac{1}{2}}\Big(\frac{2\pi|\tau|}{\sqrt{a_{2,i}}}\Big).$$

Here, $K_\nu(\cdot)$ denotes the modified Bessel function of the second kind, and $\Gamma(\cdot)$ denotes the Euler gamma function. The parameter matrices $\mathbf{e} \in \mathbb{R}^{N_v \times 3}$ and $\mathbf{a} \in \mathbb{R}^{N_v \times 4}$ describe the amplitude, temporal bandwidth, smoothness, and (for alpha) peak frequency of the respective processes. To promote sparsity in the cortical distribution of spectral components, we impose Group Lasso priors [9], where each row of these matrices defines a group. This ensures that if a particular cortical region exhibits zero power in its spectral components, all corresponding spectral parameters in that region are set to zero. The Group Lasso prior is implemented using an exponential prior $\exp(\cdot\,|\,\cdot)$ with the matrix $\ell_{2,1}$-norm:

$$\|\mathbf{U}\|_{2,1} = \sum_{i=1}^{N_v} \|\mathbf{U}_{i,:}\|_2.$$

The forward model parameters are divided into two categories. The first category includes the spectral component (SC) parameters:

$$\mathbf{x} = \Big(\mathrm{vec}_r(\mathbf{e})^\top, \mathrm{vec}_r(\mathbf{a})^\top, (\boldsymbol{\sigma}^2)^\top\Big)^\top \in \mathbb{R}^{7N_v + N_\omega},$$

where $\mathrm{vec}_r(\cdot)$ denotes row-wise vectorization, and $\boldsymbol{\sigma}^2 = (\sigma_1^2, \ldots, \sigma_{N_\omega}^2)^\top$ represents the power of the sensor noise process $\mathbf{n}_t^v$ across frequencies. The second category comprises the structural modulation parameters: $\mathbf{w} = \Big(w_C, w_D\Big)^\top$, which modulate the anatomical connectivity and conduction delays. The hyperparameters $\boldsymbol{\lambda} = (\lambda_1, \lambda_2, \lambda_3)^\top$ regularize only the SC sparsity terms.

3 Spectral Densities of the Voxel-Wise Hida-Matérn Processes

Theorem 1 (Spectral Densities of Voxel-Wise Gaussian Processes with Hida-Matérn Kernels)**.** *Let the voxel-wise stochastic innovations $\mathbf{u}_t^\eta \in \mathbb{R}^{N_v}$, with $\eta \in \{\xi, \alpha\}$, be modeled as zero-mean Gaussian processes governed by Hida-Matérn covariance functions:*

$$\mathbf{u}_t^\eta \sim \mathcal{GP}\big(\mathbf{0}, \mathbf{HM}^\eta(\tau)\big).$$

The covariance functions are defined as follows. For the aperiodic component:

$$\mathbf{HM}_{ik}^\xi(\tau) = \delta_{i,k}\, \frac{2\sqrt{\pi} e_{1,i}}{\sqrt{e_{2,i}}\,\Gamma(e_{3,i})} \left(\frac{\pi|\tau|}{\sqrt{e_{2,i}}}\right)^{e_{3,i}-\frac{1}{2}} K_{e_{3,i}-\frac{1}{2}}\Big(\frac{2\pi|\tau|}{\sqrt{e_{2,i}}}\Big).$$

For the alpha component:

$$\mathbf{HM}_{ik}^\alpha(\tau) = \delta_{i,k}\, \cos(2\pi a_{4,i}\tau)\, \frac{2\sqrt{\pi} a_{1,i}}{\sqrt{a_{2,i}}\,\Gamma(a_{3,i})} \left(\frac{\pi|\tau|}{\sqrt{a_{2,i}}}\right)^{a_{3,i}-\frac{1}{2}} K_{a_{3,i}-\frac{1}{2}}\Big(\frac{2\pi|\tau|}{\sqrt{a_{2,i}}}\Big).$$

Then, the corresponding power spectral densities at voxel i coincide with the parametric forms:

$$\boldsymbol{\xi}_{\omega,i} = \psi\big(\omega \,|\, e_{1,i},\, e_{2,i},\, e_{3,i},\, 0\big)$$

and

$$\boldsymbol{\alpha}_{\omega,i} = \frac{1}{2}\psi\big(\omega \,|\, a_{1,i},\, a_{2,i},\, a_{3,i},\, a_{4,i}\big) + \frac{1}{2}\psi\big(\omega \,|\, a_{1,i},\, a_{2,i},\, a_{3,i},\, -a_{4,i}\big),$$

where

$$\psi(\omega \mid A, B, E, F) = \frac{A}{\big(1 + B(\omega - F)^2\big)^E}.$$

Proof. Since $\mathbf{u}_t^\eta$ is modeled as a zero-mean Gaussian process with stationary covariance $\mathbf{HM}^\eta(\tau)$, the power spectral density of each univariate process $u_{t,i}^\eta$ is given by the Fourier transform:

$$S_i^\eta(\omega) = \int_{-\infty}^{\infty} \mathbf{HM}_{ii}^\eta(\tau)\, e^{-i2\pi\omega\tau}\, d\tau.$$

For $\eta = \xi$, substituting the definition of $\mathbf{HM}_{ii}^\xi(\tau)$ yields:

$$S_i^\xi(\omega) = \frac{2\sqrt{\pi}e_{1,i}}{\sqrt{e_{2,i}}\,\Gamma(e_{3,i})}\int_0^\infty \left(\frac{\pi\tau}{\sqrt{e_{2,i}}}\right)^{e_{3,i}-\frac{1}{2}} K_{e_{3,i}-\frac{1}{2}}\Big(\frac{2\pi\tau}{\sqrt{e_{2,i}}}\Big)\cos(2\pi\omega\tau)\, d\tau.$$

Applying the Weber–Schafheitlin integral (Hankel transform):

$$\int_0^\infty x^\mu K_\mu(\lambda x)\cos(\rho x)\, dx = \sqrt{\frac{\pi}{2}}\frac{\Gamma(\mu + \frac{1}{2})}{(\lambda^2 + \rho^2)^{\mu+\frac{1}{2}}},$$

where $\mu = e_{3,i} - \frac{1}{2}$, $\lambda = \frac{2\pi}{\sqrt{e_{2,i}}}$, and $\rho = 2\pi\omega$, we obtain:

$$S_i^\xi(\omega) = \frac{e_{1,i}}{\big(1 + e_{2,i}\,\omega^2\big)^{e_{3,i}}} = \boldsymbol{\xi}_{\omega,i}.$$

For $\eta = \alpha$, the covariance includes a cosine modulation:

$$\mathbf{HM}_{ii}^\alpha(\tau) = \cos(2\pi a_{4,i}\tau)\,\frac{2\sqrt{\pi}a_{1,i}}{\sqrt{a_{2,i}}\,\Gamma(a_{3,i})}\left(\frac{\pi\tau}{\sqrt{a_{2,i}}}\right)^{a_{3,i}-\frac{1}{2}} K_{a_{3,i}-\frac{1}{2}}\Big(\frac{2\pi\tau}{\sqrt{a_{2,i}}}\Big).$$

Expanding the product:

$$\cos(2\pi a_{4,i}\tau)\, e^{-i2\pi\omega\tau} = \frac{1}{2}\Big(e^{-i2\pi(\omega-a_{4,i})\tau} + e^{-i2\pi(\omega+a_{4,i})\tau}\Big).$$

Hence:

$$S_i^\alpha(\omega) = \frac{1}{2}\int_{-\infty}^{\infty}\mathcal{M}_i^\alpha(\tau)\, e^{-i2\pi(\omega-a_{4,i})\tau}\, d\tau + \frac{1}{2}\int_{-\infty}^{\infty}\mathcal{M}_i^\alpha(\tau)\, e^{-i2\pi(\omega+a_{4,i})\tau}\, d\tau.$$

For each integral, we apply the Weber–Schafheitlin integral *again*, now shifted by the respective center frequencies, giving:

$$\frac{a_{1,i}}{\big(1 + a_{2,i}(\omega \mp a_{4,i})^2\big)^{a_{3,i}}}.$$

Thus:

$$S_i^\alpha(\omega) = \frac{1}{2}\psi\big(\omega \,|\, a_{1,i}, a_{2,i}, a_{3,i}, a_{4,i}\big) + \frac{1}{2}\psi\big(\omega \,|\, a_{1,i}, a_{2,i}, a_{3,i}, -a_{4,i}\big) = \boldsymbol{\alpha}_{\omega,i}.$$

This concludes the proof. □

4 Derivation of the Frequency-Domain MAP Estimator for ξ-αNET

We begin by considering the ξ-αNET generative model in the time domain:

$$\mathbf{v}_t = \mathbf{K}\,\mathbf{j}_t + \mathbf{n}_t^v,$$

where $\mathbf{v}_t$ denotes EEG signals at the sensors, $\mathbf{K}$ is the leadfield matrix, and $\mathbf{n}_t^v$ is additive sensor noise. The source current $\mathbf{j}_t$ decomposes into an aperiodic background component and an oscillatory alpha component:

$$\mathbf{j}_t = \mathbf{j}_t^{\xi} + \mathbf{j}_t^{\alpha}.$$

Both components evolve through the same linear network structure:

$$\mathbf{j}_t^{\eta} = (\mathbf{A} \circledast \mathbf{j}^{\eta})_t + \mathbf{u}_t^{\eta}, \quad \eta \in \{\xi, \alpha\}.$$

The connectivity operator $\mathbf{A}_\tau$ encodes effective coupling between sources at discrete conduction delays τ:

$$\mathbf{A}_\tau = w_C\, \bar{\mathbf{C}} \odot \delta(\tau - w_D\, \bar{\mathbf{D}}),$$

where $\bar{\mathbf{C}}$ is the baseline anatomical connectivity matrix, $\bar{\mathbf{D}}$ is the baseline conduction delay matrix, and $\delta(\cdot)$ is the Dirac delta function applied elementwise. Applying the Fourier transform yields the frequency-domain representation:

$$\begin{aligned}
\mathbf{v}_\omega &= \mathbf{K}\,\mathbf{j}_\omega + \mathbf{n}_\omega^v, \\
\mathbf{j}_\omega &= \mathbf{j}_\omega^{\xi} + \mathbf{j}_\omega^{\alpha}, \\
\mathbf{j}_\omega^{\eta} &= (\mathbf{I}_{N_v} - \mathbf{A}_\omega)^{-1}\, \mathbf{u}_\omega^{\eta}, \quad \eta \in \{\xi, \alpha\}, \\
\mathbf{A}_\omega &= w_C\, \bar{\mathbf{C}} \odot \exp\bigl(-2\pi i \omega w_D\, \bar{\mathbf{D}}\bigr).
\end{aligned}$$

Here the exponential is applied elementwise. The innovation processes $\mathbf{u}_t^{\xi}$ and $\mathbf{u}_t^{\alpha}$ are modeled as zero-mean Gaussian processes governed by Hida-Matérn covariance functions:

$$\mathbf{u}_t^{\eta} \sim \mathcal{GP}\bigl(\mathbf{0}, \mathbf{HM}^{\eta}(\tau)\bigr).$$

By the established theorem, their frequency-domain representations are complex-valued Gaussian random variables with diagonal covariance:

$$\begin{aligned}
\mathbf{u}_\omega^{\xi} &\sim \mathcal{CN}\bigl(\mathbf{0}, \mathrm{diag}(\boldsymbol{\xi}_\omega)\bigr), \\
\mathbf{u}_\omega^{\alpha} &\sim \mathcal{CN}\bigl(\mathbf{0}, \mathrm{diag}(\boldsymbol{\alpha}_\omega)\bigr),
\end{aligned}$$

where the corresponding power spectral densities are given by:

$$\boldsymbol{\xi}_{\omega,i} = \psi(\omega \,|\, e_{1,i}, e_{2,i}, e_{3,i}, 0),$$

$$\boldsymbol{\alpha}_{\omega,i} = \frac{1}{2}\psi(\omega \,|\, a_{1,i}, a_{2,i}, a_{3,i}, a_{4,i}) + \frac{1}{2}\psi(\omega \,|\, a_{1,i}, a_{2,i}, a_{3,i}, -a_{4,i}).$$

The observation noise process is also modeled as a zero-mean Gaussian process with covariance:

$$\mathbf{n}_t^v \sim \mathcal{GP}\bigl(\mathbf{0}, \sigma_\tau^2 \mathbf{I}_{N_c} \delta(\tau)\bigr),$$

yielding in the frequency domain:

$$\mathbf{n}_\omega^v \sim \mathcal{CN}\bigl(\mathbf{0}, \sigma_\omega^2 \mathbf{I}_{N_c}\bigr).$$

Combining these, the observation equation in the frequency domain becomes:

$$\mathbf{v}_\omega = \mathbf{T}_\omega\bigl(\mathbf{u}_\omega^{\xi} + \mathbf{u}_\omega^{\alpha}\bigr) + \mathbf{n}_\omega^v,$$

where the transfer operator is:

$$\mathbf{T}_\omega = \mathbf{K}\left(\mathbf{I}_{N_v} - w_C\, \bar{\mathbf{C}} \odot \exp\bigl(-2\pi i \omega w_D\, \bar{\mathbf{D}}\bigr)\right)^{-1}.$$

Since the innovation processes are independent across components, the covariance of the observed data is:

$$\boldsymbol{\Sigma}_\omega^v = \sigma_\omega^2 \mathbf{I}_{N_c} + \mathbf{T}_\omega\, \mathrm{diag}\bigl(\boldsymbol{\xi}_\omega + \boldsymbol{\alpha}_\omega\bigr)\, \mathbf{T}_\omega^{\dagger}.$$

Here, $\boldsymbol{\xi}_\omega$ and $\boldsymbol{\alpha}_\omega$ denote the parametric source spectra evaluated at frequency ω, parameterized by vectors $\mathbf{e}$ and $\mathbf{a}$. For each subject j, the empirical cross-spectrum $\mathbf{S}_\omega^{(j)}$ provides an unbiased estimate of $\boldsymbol{\Sigma}_\omega^v$ and is approximately complex Wishart distributed. The negative log-likelihood is:

$$-\log p(\mathbf{S}_\omega^{(j)} \,|\, \boldsymbol{\Sigma}_\omega^v) \propto \log|\boldsymbol{\Sigma}_\omega^v| + \mathrm{tr}\Bigl((\boldsymbol{\Sigma}_\omega^v)^{-1}\mathbf{S}_\omega^{(j)}\Bigr).$$

Introducing the precision matrix:

$$\boldsymbol{\Omega}_\omega = \left(\boldsymbol{\Sigma}_\omega^v\right)^{-1},$$

the likelihood can be rewritten as:

$$-\log p(\mathbf{S}_\omega^{(j)} \mid \boldsymbol{\Omega}_\omega) \propto -\log|\boldsymbol{\Omega}_\omega| + \mathrm{tr}\big(\mathbf{S}_\omega^{(j)}\boldsymbol{\Omega}_\omega\big).$$

Summing over all frequencies yields the total negative log-likelihood:

$$f = \sum_\omega \Big(-\log|\boldsymbol{\Omega}_\omega| + \mathrm{tr}\big(\mathbf{S}_\omega^{(j)}\boldsymbol{\Omega}_\omega\big)\Big).$$

We impose sparsity-promoting priors over $\mathbf{e}$ and $\mathbf{a}$:

$$p(\mathbf{e}) \propto \exp(-\lambda_2\|\mathbf{e}\|_{2,1}), \quad p(\mathbf{a}) \propto \exp(-\lambda_3\|\mathbf{a}\|_{2,1}),$$

and a Gaussian prior over the noise variance vector:

$$p(\boldsymbol{\sigma}^2) \propto \exp(-\lambda_1\|\boldsymbol{\sigma}^2\|_2^2).$$

Thus, the negative log-prior is:

$$g = \lambda_1\|\boldsymbol{\sigma}^2\|_2^2 + \lambda_2\|\mathbf{e}\|_{2,1} + \lambda_3\|\mathbf{a}\|_{2,1}.$$

The resulting Maximum A Posteriori (MAP) optimization problem is:

$$\big(\mathbf{x}^{(j)}, \mathbf{w}^{(j)}\big) = \arg\min_{\mathbf{x},\mathbf{w}>0} F\big(\mathbf{S}^{(j)} \mid \mathbf{x}, \mathbf{w}\big),$$

where:

$$\begin{aligned}
F\big(\mathbf{S}^{(j)} \mid \mathbf{x}, \mathbf{w}\big) &= f + g, \\
f &= \sum_\omega \Big(-\log|\boldsymbol{\Omega}_\omega| + \mathrm{tr}\big(\mathbf{S}_\omega^{(j)}\boldsymbol{\Omega}_\omega\big)\Big), \\
g &= \lambda_1\|\boldsymbol{\sigma}^2\|_2^2 + \lambda_2\|\mathbf{e}\|_{2,1} + \lambda_3\|\mathbf{a}\|_{2,1}, \\
\boldsymbol{\Omega}_\omega &= \Big(\sigma_\omega^2\mathbf{I}_{N_c} + \mathbf{T}_\omega\,\mathrm{diag}\big(\boldsymbol{\xi}_\omega + \boldsymbol{\alpha}_\omega\big)\mathbf{T}_\omega^\dagger\Big)^{-1}, \\
\mathbf{T}_\omega &= \mathbf{K}\Big(\mathbf{I}_{N_v} - w_C\,\bar{\mathbf{C}} \odot \exp\big(-2\pi i\omega w_D\,\bar{\mathbf{D}}\big)\Big)^{-1}.
\end{aligned}$$

5 Gradient Derivation (Score Function): $\partial_{\sigma_\omega^2} f$ and $\partial_{a_{ij}} f$

We provide a detailed derivation of the partial derivatives of the smooth part of the objective function f with respect to the noise variance σ^2 and the spectral parameters a_{ij}. Recall that

$$f = \sum_\omega \left(-\log|\boldsymbol{\Omega}_\omega| + \mathrm{tr}\left(\mathbf{S}_\omega\boldsymbol{\Omega}_\omega\right)\right),$$

where $\boldsymbol{\Omega}_\omega$ is the precision matrix at frequency ω and $\mathbf{S}_\omega$ is the empirical cross-spectrum.

1. Derivation of $\partial_{\sigma_\omega^2} f$

We first compute the differential of $\boldsymbol{\Omega}_\omega$ with respect to σ_ω^2. By definition, the precision matrix satisfies

$$\boldsymbol{\Omega}_\omega = \mathbf{R}_\omega^{-1},$$

where

$$\mathbf{R}_\omega = \sigma_\omega^2\mathbf{I}_{N_c} + \mathbf{T}_\omega\mathrm{diag}(\boldsymbol{\xi}_\omega + \boldsymbol{\alpha}_\omega)\mathbf{T}_\omega^\dagger,$$

and $\mathbf{T}_\omega$ is the transfer operator at frequency ω.The differential of the inverse of a matrix gives

$$d_{\sigma_\omega^2}\boldsymbol{\Omega}_\omega = -\boldsymbol{\Omega}_\omega(d_{\sigma_\omega^2}\mathbf{R}_\omega)\boldsymbol{\Omega}_\omega.$$

Since σ_ω^2 appears only in the term $\sigma_\omega^2\mathbf{I}_{N_c}$, the differential of $\mathbf{R}_\omega$ with respect to σ_ω^2 is simply

$$d_{\sigma_\omega^2}\mathbf{R}_\omega = \mathbf{I}_{N_c} d\sigma_\omega^2.$$

Substituting into the previous expression, we obtain

$$d_{\sigma_\omega^2}\mathbf{\Omega}_\omega = -\mathbf{\Omega}_\omega^2 d\sigma_\omega^2.$$

We now compute the differential of each term in f. First, for the log-determinant term, we use the standard matrix differential identity

$$d\log|\mathbf{A}| = \mathrm{tr}(\mathbf{A}^{-1}d\mathbf{A}),$$

thus

$$d_{\sigma_\omega^2}\left(-\log|\mathbf{\Omega}_\omega|\right) = -\mathrm{tr}\left(\mathbf{\Omega}_\omega^{-1}d_{\sigma_\omega^2}\mathbf{\Omega}_\omega\right).$$

Substituting the expression for $d_{\sigma_\omega^2}\mathbf{\Omega}_\omega$, this becomes

$$d_{\sigma_\omega^2}\left(-\log|\mathbf{\Omega}_\omega|\right) = +\mathrm{tr}\left(\mathbf{\Omega}_\omega d_{\sigma_\omega^2}\mathbf{R}_\omega\right),$$

and therefore

$$d_{\sigma_\omega^2}\left(-\log|\mathbf{\Omega}_\omega|\right) = \mathrm{tr}(\mathbf{\Omega}_\omega)d\sigma_\omega^2.$$

Next, the differential of the trace term $\mathrm{tr}(\mathbf{S}_\omega\mathbf{\Omega}_\omega)$ is

$$d_{\sigma_\omega^2}\mathrm{tr}(\mathbf{S}_\omega\mathbf{\Omega}_\omega) = \mathrm{tr}(\mathbf{S}_\omega d_{\sigma_\omega^2}\mathbf{\Omega}_\omega),$$

substituting again the expression for $d_{\sigma_\omega^2}\mathbf{\Omega}_\omega$ yields

$$d_{\sigma_\omega^2}\mathrm{tr}(\mathbf{S}_\omega\mathbf{\Omega}_\omega) = -\mathrm{tr}(\mathbf{S}_\omega\mathbf{\Omega}_\omega^2)d\sigma_\omega^2.$$

Combining the two differentials, we find

$$d_{\sigma_\omega^2}\left(-\log|\mathbf{\Omega}_\omega| + \mathrm{tr}(\mathbf{S}_\omega\mathbf{\Omega}_\omega)\right) = \mathrm{tr}\left((\mathbf{I}_{N_c} - \mathbf{S}_\omega\mathbf{\Omega}_\omega)\mathbf{\Omega}_\omega\right)d\sigma_\omega^2.$$

Therefore, the partial derivative of f with respect to σ_ω^2 is

$$\frac{\partial f}{\partial\sigma_\omega^2} = \mathrm{tr}\left(\mathbf{\Omega}_\omega(\mathbf{I}_{N_c} - \mathbf{S}_\omega\mathbf{\Omega}_\omega)\right).$$

2. Derivation of $\partial_{a_{ij}}f$

We now compute the derivative of f with respect to a spectral parameter a_{ij}. Since only $\boldsymbol{\alpha}_\omega$ depends on a_{ij}, and only at the i-th entry, we first compute the differential of $\mathbf{R}_\omega$:

$$d_{a_{ij}}\mathbf{R}_\omega = \mathbf{T}_\omega\mathbf{E}_i\left(\partial_{a_{ij}}\boldsymbol{\alpha}_{\omega,i}\right)\mathbf{T}_\omega^\dagger da_{ij},$$

where $\mathbf{E}_i$ is the diagonal matrix with 1 at the i-th diagonal entry and zeros elsewhere, and $\partial_{a_{ij}}\boldsymbol{\alpha}_{\omega,i}$ denotes the partial derivative of $\boldsymbol{\alpha}_{\omega,i}$ with respect to a_{ij}. Applying again the formula for the differential of a matrix inverse, we obtain

$$d_{a_{ij}}\mathbf{\Omega}_\omega = -\mathbf{\Omega}_\omega d_{a_{ij}}\mathbf{R}_\omega\mathbf{\Omega}_\omega.$$

Substituting the expression for $d_{a_{ij}}\mathbf{R}_\omega$, we have

$$d_{a_{ij}}\mathbf{\Omega}_\omega = -\mathbf{\Omega}_\omega\mathbf{T}_\omega\mathbf{E}_i\left(\partial_{a_{ij}}\boldsymbol{\alpha}_{\omega,i}\right)\mathbf{T}_\omega^\dagger\mathbf{\Omega}_\omega da_{ij}.$$

The differential of the log-determinant term is

$$d_{a_{ij}}\left(-\log|\mathbf{\Omega}_\omega|\right) = -\mathrm{tr}\left(\mathbf{\Omega}_\omega^{-1}d_{a_{ij}}\mathbf{\Omega}_\omega\right),$$

thus

$$d_{a_{ij}}\left(-\log|\mathbf{\Omega}_\omega|\right) = \mathrm{tr}\left(\mathbf{\Omega}_\omega d_{a_{ij}}\mathbf{R}_\omega\right).$$

Substituting the form of $d_{a_{ij}}\mathbf{R}_\omega$, we find

$$d_{a_{ij}}\left(-\log|\mathbf{\Omega}_\omega|\right) = \mathrm{tr}\left(\mathbf{T}_\omega^\dagger\mathbf{\Omega}_\omega\mathbf{T}_\omega\mathbf{E}_i\right)\left(\partial_{a_{ij}}\boldsymbol{\alpha}_{\omega,i}\right)da_{ij}.$$

Similarly, the differential of the trace term is

$$d_{a_{ij}}\mathrm{tr}(\mathbf{S}_\omega\mathbf{\Omega}_\omega) = \mathrm{tr}(\mathbf{S}_\omega d_{a_{ij}}\mathbf{\Omega}_\omega),$$

thus

$$d_{a_{ij}}\mathrm{tr}(\mathbf{S}_\omega\mathbf{\Omega}_\omega) = -\mathrm{tr}\left(\mathbf{T}_\omega^\dagger\mathbf{\Omega}_\omega\mathbf{S}_\omega\mathbf{\Omega}_\omega\mathbf{T}_\omega\mathbf{E}_i\right)\left(\partial_{a_{ij}}\boldsymbol{\alpha}_{\omega,i}\right)da_{ij}.$$

Adding both contributions, the total differential is

$$d_{a_{ij}}f = \sum_\omega \mathrm{tr}\left(\mathbf{T}_\omega^\dagger\mathbf{\Omega}_\omega(\mathbf{I}_{N_c} - \mathbf{S}_\omega\mathbf{\Omega}_\omega)\mathbf{T}_\omega\mathbf{E}_i\right)\left(\partial_{a_{ij}}\boldsymbol{\alpha}_{\omega,i}\right)da_{ij}.$$

Thus, the partial derivative of f with respect to a_{ij} is

$$\frac{\partial f}{\partial a_{ij}} = \sum_\omega\left(\mathbf{T}_\omega^\dagger\mathbf{\Omega}_\omega(\mathbf{I}_{N_c} - \mathbf{S}_\omega\mathbf{\Omega}_\omega)\mathbf{T}_\omega\right)_{ii}\partial_{a_{ij}}\boldsymbol{\alpha}_{\omega,i}.$$

6 ξ-αNET Spectral Components Search Space

As demonstrated in Supplementary Materials A, the ξ-αNET generative model predicts the sensor-level cross-spectrum of EEG signals at frequency ω according to the forward model

$$\mathbf{\Sigma}_{\omega}^{v} = \sigma_{\omega}^{2}\mathbf{I}_{N_c} + \mathbf{T}_{\omega}\,\mathrm{diag}\left(\boldsymbol{\xi}_{\omega} + \boldsymbol{\alpha}_{\omega}\right)\mathbf{T}_{\omega}^{\dagger},$$

where $\sigma_{\omega}^{2}\mathbf{I}_{N_c}$ represents sensor noise, $\mathbf{T}_{\omega}$ denotes the frequency-dependent forward operator, and the diagonal term models voxelwise latent spectral activity, composed of a smooth aperiodic process $\boldsymbol{\xi}_{\omega}$ and a peaked oscillatory process $\boldsymbol{\alpha}_{\omega}$. To estimate the latent spectral parameters from empirical observations, we first project the measured cross-spectrum $\mathbf{S}_{\omega}$ into the latent space by applying a minimum-norm back-projection. Specifically, we compute

$$\mathbf{s}_{\omega} = \mathrm{diag}\left(\mathbf{T}_{\omega}^{+}\mathbf{S}_{\omega}\left(\mathbf{T}_{\omega}^{+}\right)^{\dagger}\right),$$

where $\mathbf{T}_{\omega}^{+}$ denotes the Moore–Penrose pseudoinverse of the forward operator. The resulting $\mathbf{s}_{\omega} \in \mathbb{R}^{N_v \times 1}$ contains the estimated power spectra for each voxel at frequency ω. It is essential to note that this latent space projection differs from classical source localization, as it represents the domain where the underlying Xi and Alpha processes are assumed to be generated.

Following the latent projection, we proceed to voxel-wise estimation of the Xi–Alpha parameters. For each voxel independently, the aperiodic parameters $\mathbf{e} = (e_1, e_2, e_3)$ and the alpha parameters $\mathbf{a} = (a_1, a_2, a_3, a_4)$ are obtained by solving the nonlinear least-squares problem

$$(\widehat{\mathbf{e}}, \widehat{\mathbf{a}}) = \arg\min_{\mathbf{e},\mathbf{a}} \sum_{\omega} \left\| \log \mathbf{s}_{\omega} - \log\left(\boldsymbol{\xi}_{\omega} + \boldsymbol{\alpha}_{\omega}\right) \right\|_2^2.$$

The logarithmic transformation in the cost function balances the contributions of low- and high-power frequencies, stabilizing the optimization numerically, while preserving the nonlinearity of the mapping from parameters to spectra. The search space for the voxelwise optimization is carefully constructed to ensure physiological plausibility and robust convergence. The baseline term e_1 is fixed to the first value of the latent spectrum, $e_1 = \mathbf{s}_{\omega}(1)$, reflecting the power at the lowest frequency. The remaining parameters are randomly initialized within empirically motivated bounds: e_2 is initialized uniformly between 0 and 1, e_3 between 1 and 2.5, a_1 between 0.5 and 1 times the maximum value of $\mathbf{s}_{\omega}$, a_2 between 0 and 0.5, a_3 between 2 and 5, and a_4 between 7 and 13 Hz. The optimization is constrained within the bounds $e_1, e_2, e_3, a_1, a_2, a_3 > 0$ and $7 \leq a_4 \leq 13$ Hz, ensuring that the alpha peak remains within the expected physiological range [20, 19]. To mitigate the risk of local minima due to the nonconvexity of the problem, the voxelwise fitting procedure is repeated with 30 different random initializations for each voxel. For each initialization, the optimization is performed independently, and the parameter set that achieves the lowest residual error is selected as the voxelwise estimate $(\widehat{\mathbf{e}}, \widehat{\mathbf{a}})$. After fitting across all voxels, the variability of the parameter estimates is quantified by computing the empirical variance of each parameter across the voxel ensemble. This variance reflects the natural dispersion of spectral characteristics over the cortical surface. The final global search space for subsequent group-level or hierarchical modeling steps in the ξ-αNET framework is then defined by scaling this voxelwise variance by a factor of two:

$$\text{Search Space Width} = 2 \times \mathrm{Var}(\widehat{\mathbf{e}}, \widehat{\mathbf{a}}).$$

Thus, the search space becomes data-adaptive, ensuring that the global optimization remains both flexible and constrained according to the physiological variability captured during the voxelwise estimation.

7 Selection of the Regularization Space

The estimation of the parameters in the ξ-αNET model is formulated as a maximum a posteriori (MAP) optimization problem, as discussed in Supplementary Material A. This problem is solved using a Stochastic FISTA algorithm, which relies on a proximal gradient method. A key step in this optimization procedure is the selection of an appropriate regularization space, determined by the parameters λ_1, λ_2, and λ_3. To define this space rigorously, we seek the minimum regularization values such that, for any λ exceeding these thresholds, the proximal gradient method produces a trivial solution where all parameters are zero [2, 8]. Formally, the condition for this regime is expressed as

$$\mathrm{prox}_g\left(\nabla_{\mathbf{x}} f(\mathbf{0}, \mathbf{w})\right) = \mathbf{0},$$

where $f(\mathbf{x}, \mathbf{w})$ denotes the smooth part of the MAP objective, $g(\mathbf{x})$ denotes the nonsmooth regularization term, $\mathbf{x} = \left(\mathrm{vec}_r(\mathbf{e})^\top, \mathrm{vec}_r(\mathbf{a})^\top, (\boldsymbol{\sigma}^2)^\top\right)^\top \in \mathbb{R}^{7N_v+N_\omega}$ collects the parameters to be estimated, and $\mathbf{w}$ represents fixed structural weights. Decomposing the regularization into its separate components, this global condition expands into three independent proximal operator conditions, one for each group of parameters: the noise variances $\boldsymbol{\sigma}^2$, the Xi parameters $\mathbf{e}$, and the Alpha parameters $\mathbf{a}$. For the variance parameters $\boldsymbol{\sigma}^2$, the regularization is a squared ℓ_2 norm, $\lambda_1\|\cdot\|_2^2$. The proximal operator for this penalty has a closed-form solution given by

$$\mathrm{prox}_{\lambda_1\|\cdot\|_2^2}(v) = \frac{1}{1+2\lambda_1}v,$$

where $v = \nabla_{\boldsymbol{\sigma}^2} f(\mathbf{0}, \mathbf{w})$ denotes the gradient evaluated at the origin. Requiring that the proximal operator yields zero implies that $\frac{1}{1+2\lambda_1}v = 0$. Since the gradient is in general nonzero, the only way to satisfy this condition is to ensure that the scaling factor annihilates v, leading to the threshold

$$\lambda_1^* = \|\nabla_{\boldsymbol{\sigma}^2} f(\mathbf{0}, \mathbf{w})\|_2 .$$

Turning to the Xi parameters $\mathbf{e}$, the regularization is given by the $\ell_{2,1}$ norm applied across the rows of the parameter matrix $\mathbf{e} \in \mathbb{R}^{N_v \times 3}$, where N_v is the number of voxels. The $\ell_{2,1}$ norm is defined as

$$\|\mathbf{e}\|_{2,1} = \sum_{j=1}^{N_v} \|\mathbf{e}_j\|_2 ,$$

where $\mathbf{e}_j$ denotes the j-th row of $\mathbf{e}$. The proximal operator for this penalty acts row-by-row via a block soft-thresholding rule:

$$\left(\mathrm{prox}_{\lambda_2\|\cdot\|_{2,1}}(V)\right)_j = \left(1 - \frac{\lambda_2}{\|V_j\|_2}\right)_+ V_j,$$

where $V = \nabla_{\mathbf{e}} f(\mathbf{0}, \mathbf{w})$ is the gradient matrix and $(\cdot)_+$ denotes the positive part operator. Setting the proximal operator to zero requires that for each row j, either the gradient norm $\|V_j\|_2$ is smaller than or equal to λ_2, or the soft-thresholding reduces V_j to zero. Therefore, to ensure that all rows are simultaneously zeroed out, λ_2 must be greater than or equal to the maximum ℓ_2 norm across all rows of the gradient. This yields the threshold

$$\lambda_2^* = \max_j \left\|(\nabla_{\mathbf{e}} f(\mathbf{0}, \mathbf{w}))_j\right\|_2 .$$

The same argument applies directly to the Alpha parameters $\mathbf{a} \in \mathbb{R}^{N_v \times 4}$, which are regularized using an $\ell_{2,1}$ penalty across their rows. Thus, the corresponding threshold is given by

$$\lambda_3^* = \max_j \left\|(\nabla_{\mathbf{a}} f(\mathbf{0}, \mathbf{w}))_j\right\|_2 .$$

Collecting these results, the critical regularization thresholds ensuring trivial (zero) solutions are given by

$$\lambda_1^* = \|\nabla_{\boldsymbol{\sigma}^2} f(\mathbf{0}, \mathbf{w})\|_2 , \quad \lambda_2^* = \max_j \left\|(\nabla_{\mathbf{e}} f(\mathbf{0}, \mathbf{w}))_j\right\|_2 , \quad \lambda_3^* = \max_j \left\|(\nabla_{\mathbf{a}} f(\mathbf{0}, \mathbf{w}))_j\right\|_2 .$$

These thresholds define the maximum regularization strengths allowable for each parameter group. Subsequently, regularization paths for model selection are constructed by defining grids of λ values below these critical values, ensuring that non-trivial, interpretable solutions are obtained during optimization. This construction of the regularization space guarantees that the model achieves a balance between accurate data fitting and sparsity-enforced interpretability.

8 Automatic selection of data-driven thresholds by Expectation Maximisation

The threshold-selection procedure begins with the one-dimensional sample $V = \{v_i\}_{i=1}^N$, which, in the context of the main text, represents the voxel-wise power of the SC term. All undefined entries are discarded, the vector

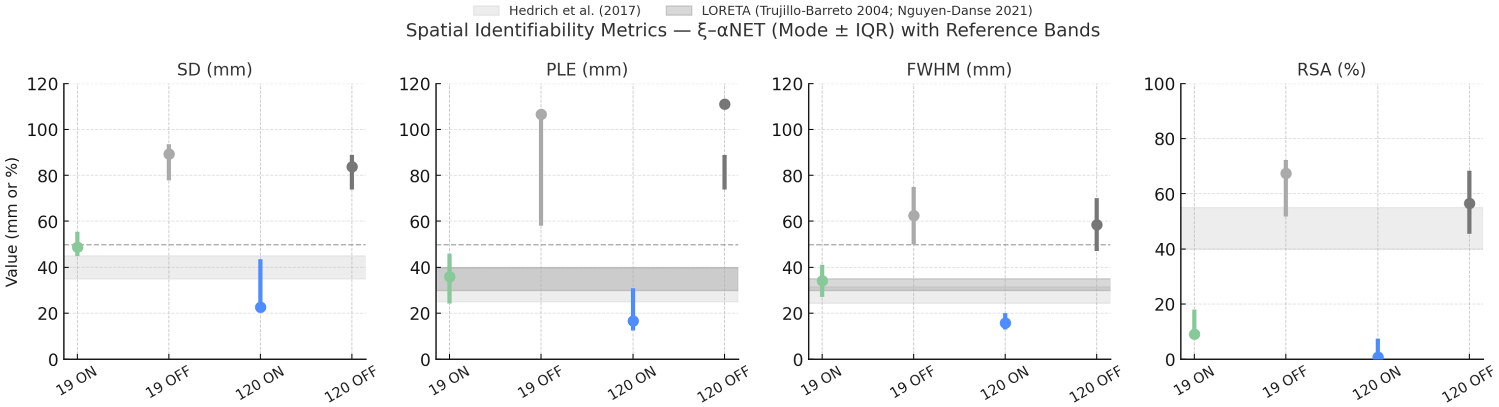

Figure 1: **Comparison of spatial identifiability metrics between ξ–αNET and hdEEG reference bands.** This figure presents the same spatial identifiability results shown in Fig. 3 (a-b) in the paper, but here the ξ–αNET estimates are directly compared against the reference intervals reported by Hedrich *et al.* [11] and other hdEEG studies (LORETA, dSPM, MNE, sLORETA; Nguyen-Danse *et al.* [18]). Even with only 19 electrodes, ξ–αNET achieves spatial dispersion (SD) and point-spread metrics (PLE, FWHM) within the benchmark range for 256-channel hdEEG systems, while yielding markedly lower RSA values—indicating a substantial reduction of spurious activity. With 120 channels, all metrics (SD, PLE, FWHM, RSA) improve further, fully matching or surpassing the hdEEG reference bands.

is flattened, and values whose magnitude falls below 10^{-10} are removed to prevent numerical pathologies. The cleaned data are then modelled as a two-component Gaussian mixture

$$p(v \mid \Theta) = \sum_{k=1}^{2} \pi_k \, \mathcal{N}\big(v \mid \mu_k, \sigma_k^2\big), \quad \Theta = \{\pi_k, \mu_k, \sigma_k^2\}_{k=1}^{2},$$

where $\pi_k > 0$ and $\pi_1 + \pi_2 = 1$ are the mixing proportions, μ_k the component means and σ_k^2 the variances. Maximum-likelihood estimates $\widehat{\Theta}$ are obtained with the expectation–maximisation (EM) algorithm, which is restarted twenty times from random initial conditions; the solution with the highest final log-likelihood is retained. To avoid singular covariances, a small ridge constant 10^{-3} is added to each estimated variance, and iteration stops when the relative improvement of the log-likelihood drops below 10^{-3} or after one thousand iterations, whichever occurs first. If EM fails to converge—an eventuality that can arise when the empirical distribution is nearly unimodal—the method falls back to the empirical 90th percentile of V. Once the mixture has been fitted, the two Gaussians are labelled so that $\mu_1 < \mu_2$ and their variances are denoted σ_1^2 and σ_2^2. The threshold is defined as the unique abscissa t^* lying between μ_1 and μ_2 at which the two unweighted densities are equal. Equating these densities yields the transcendental condition

$$\frac{(t-\mu_1)^2}{2\sigma_1^2} - \frac{(t-\mu_2)^2}{2\sigma_2^2} = \ln\big(\sigma_2/\sigma_1\big), \tag{E.1}$$

which reduces to the midpoint $(\mu_1+\mu_2)/2$ when the variances coincide. In the general, unequal-variance case, the equality of densities defines a smooth scalar function $f(t)$ whose root is sought numerically. Starting from the interval $[\mu_1, \mu_2]$, the endpoints are expanded symmetrically by successive multiples of the corresponding standard deviations until the function changes sign, after which a bracketing root-finder isolates the unique solution t^*. If a sign change cannot be produced within twenty expansions, the algorithm again reverts to the 90-th percentile fallback. The resulting value

$$t^* = \arg\{f(t) = 0\}, \tag{E.2}$$

marks the point where the likelihood of membership in either component is equal under equal variances, or where the instantaneous contribution of the two components to the overall density is identical under unequal variances. This intersection, therefore, provides a statistically principled, data-adaptive demarcation between the low-power and high-power regimes of SC. Because the procedure requires no external tuning and accommodates both symmetric and skewed empirical distributions, it furnishes an automatic and robust threshold that can be applied across all data sets analysed in the present study.

9 Resolution–matrix derivation & Ablation setup

The ξ–αNET have a nonlinear generative model that infers source parameters from scalp source cross-spectra, rather than direct time-series activity, like traditional inverse solutions. Therefore, to analyze spatial dispersion comparable to other methods of inverse solution in the literature, we linearized the model around the posterior mode to obtain equivalent forward operators. The approximate forward of the model is given by

$$\mathbf{F}_\omega = \mathbf{K}(\mathbf{I} - w_C\mathbf{C} \odot \exp(-2\pi i\omega w_D\mathbf{D}))^{-1} \tag{1}$$

where $\mathbf{K}$ its the leadfield w_C and w_D are the structural parameters over the connectivity and conduction delay matrix, that the ξ–αNET estimates. This linearization enabled computation of the analytical Resolution Matrix and point-spread-function (PSF) metrics using the same definitions and procedures as Hedrich et al., [11]. Because the actual inversion in ξ–αNET employs sparse hierarchical priors, the reported spatial dispersion values represent upper bounds to the true effective resolution achieved by the nonlinear estimator.

Following Hedrich et al., [11], we quantified: (1) Peak Localization Error (PLE $\approx$ DLE) — distance between true and reconstructed source peaks (mm); (2) Spatial Dispersion (SD) — full-width-at-half-maximum (FWHM) of the PSF (mm); (3) Ratio of Spurious Activity (RSA) — fraction of reconstructed energy outside a 75 mm geodesic radius around the true source. Fig. 1 is a comparison of spatial identifiability metrics between ξ–αNET and hdEEG reference intervals [11].

10 Test-retest reliability and cross-dataset replication

To address this, we evaluated the short-term reliability of ξ–αNET using an open-access dataset published in *Nature Scientific Data* [25, 6, 15]. This dataset includes 64-channel EEG recordings acquired during two resting conditions (eyes open and eyes closed) and three subject-driven cognitive states (memory, music, and subtraction), recorded across both short-term (within 90 minutes) and long-term (one month apart) designs. Sixty participants were recorded in three EEG sessions, each accompanied by behavioral and psychometric data, including assessments of sleep, emotion, mental health, and self-generated thoughts (mind wandering).

For our analysis, we focused on the eyes-closed condition from the first two sessions (recorded 90 minutes apart) to estimate short-term cortical test–retest reliability. The ξ–αNET model was inverted for each participant to estimate the full set of source spectral parameters—including $\mathbf{A}\alpha$ (Power), $\mathbf{B}\alpha$ (Bandwidth), $\mathbf{E}\alpha$ (Exponent), and $\mathbf{F}\alpha$ (Peak Alpha Frequency) for the α component, and $\mathbf{A}\xi$, $\mathbf{B}\xi$, $\mathbf{E}\xi$ for the aperiodic ξ component—across 8,003 cortical voxels. These voxel-level maps were projected onto the HCP-MMP1 and Yeo-7NET parcellation for statistical evaluation.

Reliability was quantified using the Intraclass Correlation Coefficient (ICC), following the two-way mixed, consistency, single-measure form ICC(3,1) described by [23], defined as

$$\text{ICC} = \frac{MS_p - MS_e}{MS_p + (d-1)MS_e}, \tag{2}$$

where $d = 2$ sessions, $n = 60$ subjects, MS_p is the mean square between subjects, and MS_e is the mean square error. For each ROI, ICC(3,1) values quantify the proportion of variance explained by consistent inter-subject differences relative to measurement error.

In contrast to the ANOVA-based ICC analysis used by [6], which assesses the significance of ICC values relative to $H_0 : \text{ICC} = 0$, our approach validated a stronger null hypothesis $H_0 : \text{ICC} \leq \rho_0$ with $\rho_0 = 0.4$. Specifically, we employed a nonparametric bootstrap ($B = 1000$ resamples) to derive 95% confidence intervals and a permutation-based test ($B = 5000$ iterations) to determine whether observed ICC values significantly exceeded this reliability threshold. For each permutation, session-2 data were randomly reassigned across subjects, yielding an empirical null distribution of ICCs shifted by ρ_0. Resulting p-values were corrected for multiple comparisons using the Benjamini–Hochberg false discovery rate (FDR) at $q_{FDR} = 0.05$.

In addition, cross-dataset replication analyses were performed to assess the generalizability of the ξ–αNET estimates. As shown in Fig. 2, the model inverted over two independent datasets—a 64-channel test–retest cohort ($N = 60$, first two sessions) and the large-scale HarMNqEEG dataset ($N = 1965$)—yielded highly consistent spatial probability atlases i.e. spatial localization gradients ($r > 0.9$, $P_{\text{spin}} < 0.01$, Computed using the Neuromaps plugin inside Brainstorm) and qualitatively similar directed connectivity patterns, with α showing predominant feedback and ξ predominant feedforward organization. These results confirm that the spatial and hierarchical features of the ξ–αNET model are reproducible across both datasets and acquisition settings.

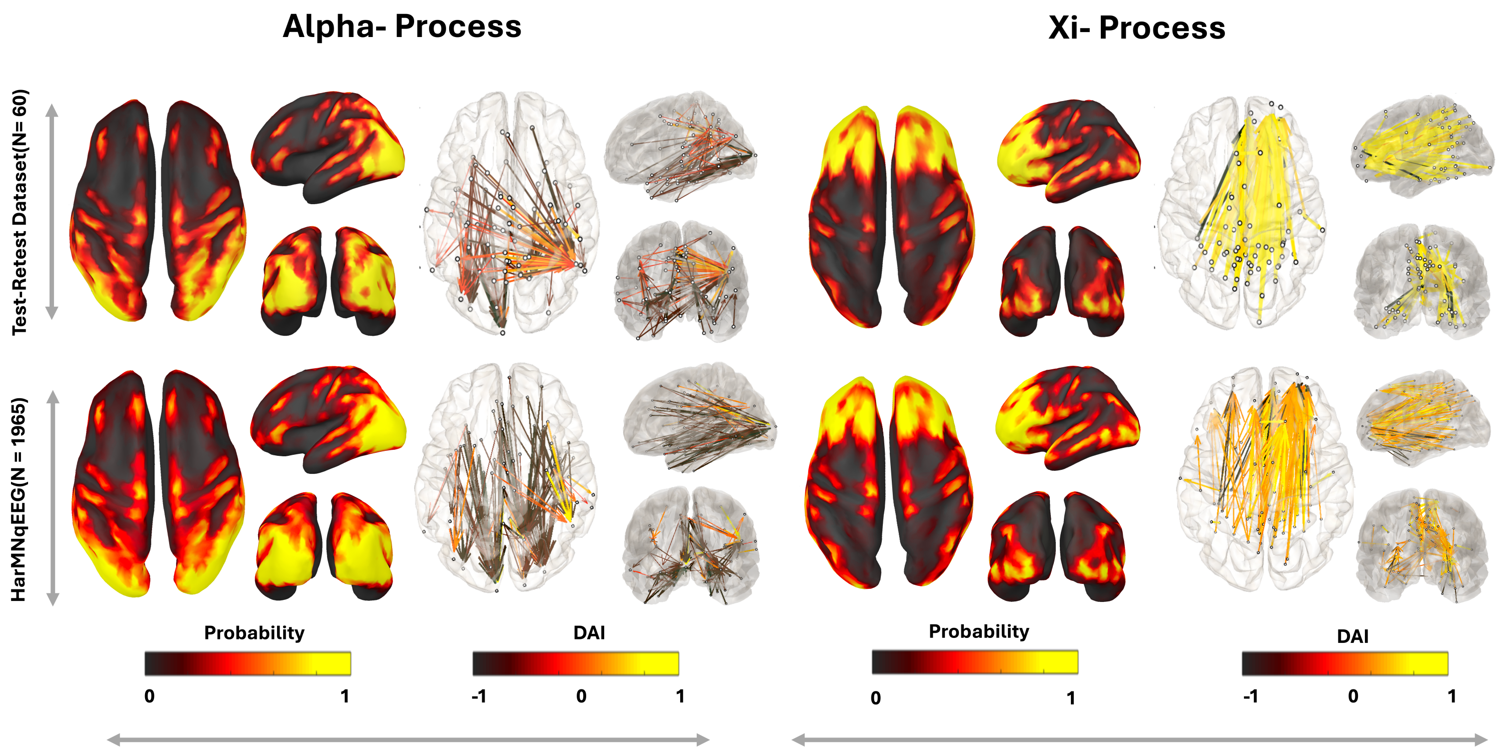

Figure 2: **Cross-dataset replication of α- and ξ-process probability atlases and directed connectivity**. The figure compares the estimated probability atlases and Spectral Granger Causality (Directional Asymmetry Index, DAI) obtained by inverting the ξ–αNET model on two independent datasets: a test–retest cohort ($N = 60$, first two sessions) and the HarMNqEEG dataset ($N = 1965$). The spatial probability maps show highly consistent topographies between datasets ($r > 0.9$, $P_{\text{spin}} < 0.01$, computed using BrainStorm Neuromaps plugin). In both datasets, the α-process displays predominant feedback connectivity, whereas the ξ-process exhibits dominant feedforward connectivity.

11 Delayed Neural Mass Model with Structural and Delay Priors

To validate the ξ–αNET model, we generated synthetic EEG data using a biophysically informed Delayed Neural Mass Model that differs structurally and generatively from the model assumed by ξ–αNET, thus avoiding the inverse crime. The cortical surface is modeled as a field of $N = 360$ neural masses, each corresponding to a region in the HCP-MMP1.0 parcellation [10].

Structural connectivity between these regions is described by the matrix $\mathbf{C} \in \mathbb{R}^{360\times360}$, derived from diffusion MRI as reported in Rosen et al. [21], while inter-regional conduction delays are specified by the matrix $\mathbf{D} \in \mathbb{R}^{360\times360}$, obtained from cortico-cortical evoked potentials (CCEPs) using the atlas of Lemarechal et al. [13]. Both atlases are defined in the same anatomical space and aligned with the HCP-MMP1.0 parcelation.

To ensure physiologically realistic spectral features, each neural mass is modeled as the sum of two coupled damped oscillators: an aperiodic component centered at 0 Hz, and an alpha-band oscillator with a frequency randomly drawn from $f_0 \sim \mathcal{U}(8, 13)$ Hz. These components follow the delayed second-order stochastic differential equations:

$$\frac{d^2u_i^{(1)}(t)}{dt^2} + 2\zeta_1\omega_1\frac{du_i^{(1)}(t)}{dt} + \omega_1^2u_i^{(1)}(t) = \sum_{j=1}^{N} C_{ij}\cdot\sigma\big(u_j^{(1)}(t-D_{ij}) + u_j^{(2)}(t-D_{ij})\big) + \eta_i^{(1)}(t)$$

$$\frac{d^2u_i^{(2)}(t)}{dt^2} + 2\zeta_2\omega_2\frac{du_i^{(2)}(t)}{dt} + \omega_2^2u_i^{(2)}(t) = \sum_{j=1}^{N} C_{ij}\cdot\sigma\big(u_j^{(1)}(t-D_{ij}) + u_j^{(2)}(t-D_{ij})\big) + \eta_i^{(2)}(t)$$

Here, $u_i^{(1)}(t)$ and $u_i^{(2)}(t)$ are the aperiodic and alpha components of the neural activity at region i, with $\omega_1 = 0$, $\omega_2 = 2\pi f_0$, ζ_1, ζ_2 the damping ratios, and $\eta_i^{(1)}(t)$, $\eta_i^{(2)}(t)$ Gaussian white noise processes. The total activity is given by $u_i(t) = u_i^{(1)}(t) + u_i^{(2)}(t)$. The nonlinearity $\sigma(x) = \frac{1}{1+e^{-x}}$ models saturation of delayed inputs. These dynamics are simulated over $T = 5$ second with 1 ms resolution and repeated over $M = 20$ trials to support

ensemble averaging. The source-level cross-spectrum tensor is computed by first applying the Fourier transform to each regional time series and then averaging across trials:

$$\mathbf{S}_\omega = \frac{1}{M}\sum_{m=1}^{M}\frac{1}{T}\hat{\mathbf{u}}^{(m)}(\omega)\hat{\mathbf{u}}^{(m)}(\omega)^*,$$

where $\hat{\mathbf{u}}^{(m)}(\omega)$ denotes the frequency-domain activity for trial m. The tensor $\mathbf{S} \in \mathbb{C}^{360\times360\times F}$ is computed across $F = 47$ frequency bins evenly spaced over the interval $[0.3, 20]$ Hz. To emulate EEG recordings, we project the simulated cortical signals to the scalp using a realistic lead field matrix $\mathbf{K} \in \mathbb{R}^{n_c\times360}$, producing:

$$\mathbf{v}(t) = \mathbf{K}\mathbf{u}(t) + \mathbf{e}(t),$$

where $\mathbf{e}(t) \sim \mathcal{N}(0, \sigma^2\mathbf{I})$ is additive Gaussian sensor noise. The corresponding sensor-level cross-spectrum is then computed as:

$$\mathbf{S}_\omega^{\text{scalp}} = \mathbf{K}\mathbf{S}_\omega\mathbf{K}^\top + \sigma^2\mathbf{I}.$$

To induce realistic spatial patterns in the simulated cortical activity, we initialized the oscillators using region-specific masks. The aperiodic component was seeded with a uniform initial condition across all ROIs, while the alpha component was initialized with higher amplitude in posterior areas (occipital, parietal, and temporal lobes). This ensures that the resulting simulated data exhibit a topographical distribution of alpha power consistent with electrophysiological recordings. These biologically grounded simulations provide realistic cortical and scalp-level cross-spectra that serve as ground truth for evaluating the accuracy of the ξ–αNET model.

12 Nonparametric estimation of the Probability Atlas

This nonparametric regression approach enabled robust estimation of conditional probabilities over age, providing spatially resolved maps of SC expression trajectories throughout the lifespan [7]:

$$\begin{aligned}
f_\xi(i,a) &= \frac{1}{Z}\sum_{k,j=1}^{N_v,N_s} K_{\mathbf{h}}\big(a-a_j,\, l_{i,k}\big)\,\mathbb{I}\big(\mathbf{A}\boldsymbol{\xi}_i^{(j)} > T_\xi\big),\\
f_\alpha(i,a) &= \frac{1}{Z}\sum_{k,j=1}^{N_v,N_s} K_{\mathbf{h}}\big(a-a_j,\, l_{i,k}\big)\,\mathbb{I}\big(\mathbf{A}\boldsymbol{\alpha}_i^{(j)} > T_\alpha\big),\\
Z(i,a) &= \sum_{k,j=1}^{N_v,N_s} K_{\mathbf{h}}\big(a-a_j,\, l_{i,k}\big),\\
K_{\mathbf{h}}(\mathbf{x}) &= \frac{9}{16}\prod_{k=1}^{2}\left(1-\frac{x_k^2}{h_k^2}\right)\mathbb{I}\left(|x_k|<h_k\right),
\end{aligned}$$

where $K_{\mathbf{h}}(\cdot)$ is the Epanechnikov kernel with a smoothing parameter $\mathbf{h} = (h_1, h_2)^\top$ selected by 10-fold cross-validation. Here, $l_{i,k}$ is the distance between vertices i and k, $Z(i,a)$ is a normalization constant, and $\mathbb{I}(\cdot)$ is an indicator function that equals one if the SC power exceeds a threshold (T_ξ or T_α). These thresholds are set automatically by fitting a mixture of two Gaussian distributions on the power of the SC through the use of Expectation Maximization, and it is defined as the point at which the two Gaussian distributions overlap (see details in Supplementary §8). From the density estimation, we compute the spatial distribution of SC as the marginal distribution across age groups.

13 Estimation of Spectral granger causality

Since ξ–αNET defines a parametric spectral factorization of source cross-spectra for each spectral process (xi and alpha) through Eqs. (5)–(6), we computed the spectral Granger causality (Geweke-Granger causality, GGC) $\mathbf{G}_{\omega,ji}$ using the formulation of Dhamala et al. [5]. For each process, we summarized the vertex-wise GGC over the HCP-MMP1 parcellation [10]. Specifically, for the alpha process we evaluated $\mathbf{G}^{\alpha}_{\omega_\alpha,j\to i}$ at the frequency within the alpha band with maximal spectral power (ω_α), and for the xi process $\mathbf{G}^{\xi}_{\omega_\xi,j\to i}$ at the corresponding peak in the delta range (ω_ξ). More specifically, we use the following formal that results from

using the equation of Dhamala et al., [5] of GGC over the spectral factorization of the cross-spectra of each spectral process in our paper Eqs. (5) and (6)

$$\mathbf{G}^{\xi}_{\omega_\xi, j\to i} = \log\left(\frac{\mathbf{S}^{\xi}_{\omega,ii}}{\mathbf{S}^{\xi}_{\omega,ii} - |\mathbf{H}^{\xi}_{\omega,ji}|^2}\right), \tag{3}$$

$$\mathbf{G}^{\alpha}_{\omega_\alpha, j\to i} = \log\left(\frac{\mathbf{S}^{\alpha}_{\omega,ii}}{\mathbf{S}^{\alpha}_{\omega,ii} - |\mathbf{H}^{\alpha}_{\omega,ji}|^2}\right). \tag{4}$$

We averaged the resulting Granger causality matrices across subjects using the Euclidean mean, both within each age group and across the full sample. The statistical significance of directed connections was assessed using a nonparametric permutation test, based on 5000 random relabelings of the regions of interest, which jointly permuted the rows and columns of the group-mean matrix at each iteration. This procedure tests the null hypothesis that the spatial organization of Granger causality is random with respect to cortical anatomy. Connections whose empirical values exceeded the 99th percentile of the permutation-derived null distribution ($\alpha = 0.01$) were deemed significant.

To determine whether significant Granger-causal connections were predominantly feedback or feedforward, we computed the Directional Asymmetry Index (DAI) in relation to the cortical hierarchy defined by the T1w/T2w myelin ratio, following Burt et al. [4]. This index distinguishes feedforward from feedback connections based on the cortical myelination gradient, which correlates with the laminar hierarchy observed in nonhuman primates. We obtained the T1w/T2w myelin map from the Human Connectome Project (S1200 release) using the Neuromaps toolbox and summarized it over the HCP-MMP1 parcellation. For each pair of regions showing significant bidirectional connectivity, we computed the DAI as:

$$\mathbf{DAI}^{\alpha}_{j\to i} = \mathrm{sign}(h_j - h_i)\frac{\mathbf{G}^{\alpha}_{\omega_\alpha, j\to i} - \mathbf{G}^{\alpha}_{\omega_\alpha, i\to j}}{\mathbf{G}^{\alpha}_{\omega_\alpha, j\to i} + \mathbf{G}^{\alpha}_{\omega_\alpha, i\to j}}, \tag{5}$$

$$\mathbf{DAI}^{\xi}_{j\to i} = \mathrm{sign}(h_j - h_i)\frac{\mathbf{G}^{\xi}_{\omega_\xi, j\to i} - \mathbf{G}^{\xi}_{\omega_\xi, i\to j}}{\mathbf{G}^{\xi}_{\omega_\xi, j\to i} + \mathbf{G}^{\xi}_{\omega_\xi, i\to j}}. \tag{6}$$

The DAI is a normalized measure ranging from -1 (feedback) to $+1$ (feedforward). This metric is illustrated in Figs. 4 (b), (f), (d), and (h). The plots reveal that the alpha and xi processes form distinct effective networks: nearly all significant alpha connections are feedback-oriented toward posterior cortical areas, whereas the xi-process connections are predominantly feedforward. This pattern remains consistent across the lifespan—alpha networks retain feedback dominance, while xi networks remain feedforward.

Finally, we conducted a permutation test to assess whether the significant Granger causality (GC) values were preferentially expressed over feedback or feedforward pathways. To evaluate the null hypothesis, the cortical hierarchy (derived from myelination values) was randomly permuted across regions, thereby generating a spatial null distribution of feedforward–feedback assignments. The test evaluated the contrasts H_0 : $\Delta_{\text{Feedback–Feedforward}}\,\mathrm{GC} > 0$ and H_0 : $\Delta_{\text{Feedforward–Feedback}}\,\mathrm{GC} > 0$ for each spectral process, replicating the analysis of Michalareas et al. [17] (their Fig. 4B). The results showed that GC values were significantly greater over feedback connections for the alpha process ($p = 0.005$) and over feedforward connections for the xi process ($p = 0.001$).

14 Voxel-wise Zero-Inflated Gaussian (ZIG) Model and Estimation Procedure

To investigate age-related variations in EEG spectral parameters estimated by the ξ–αNET model, we applied a voxel-wise Zero-Inflated Gaussian (ZIG) regression model. This model is tailored to data that are non-negative, positively skewed, and contain a high proportion of structural zeros—features commonly observed in the aperiodic amplitude $A\xi$, the narrowband alpha amplitude $A\alpha$, and the peak alpha frequency $F\alpha$ estimated at the cortical source level. Let Y_{ij} denote the spectral measurement at voxel i for subject j, and let a_j represent the subject's age. The ZIG model assumes a two-component generative process:

$$Y_{ij} \sim \pi_{ij} \cdot \delta_0 + (1 - \pi_{ij}) \cdot \mathcal{N}^+(\mu_{ij}, \sigma_i^2),$$

where δ_0 is a point mass at zero (modeling structural zeros), $\mathcal{N}^+(\mu_{ij}, \sigma_i^2)$ denotes a Gaussian distribution truncated to the positive real line, μ_{ij} is the conditional mean, σ_i^2 is the residual variance, and π_{ij} is the

zero-inflation probability. The conditional mean and zero-inflation probability are modeled as functions of chronological (unstandardized) age:

$$\mu_{ij} = \beta_{0,i} + \beta_{1,i} a_j + \beta_{2,i} a_j^2,$$

$$\operatorname{logit}(\pi_{ij}) = \gamma_{0,i} + \gamma_{1,i} a_j.$$

This formulation allows us to estimate, at each voxel, both the age-related likelihood of observing a structural zero (via π_{ij}) and the magnitude of the signal when it is present (via μ_{ij}). The quadratic term in the conditional mean enables the detection of non-monotonic trajectories, such as inverted-U patterns across the lifespan.

Estimation Procedure

To estimate the model parameters, we implemented a two-step procedure. For numerical stability and interpretability of coefficients, we standardized the age variable as

$$\tilde{a}_j = \frac{a_j - \bar{a}}{\operatorname{std}(a)}.$$

In the first step, we fit a logistic regression to the binary indicator $Z_{ij} = \mathbb{I}(Y_{ij} = 0)$, modeling the zero-inflation probability using a binomial generalized linear model:

$$\operatorname{logit}(\pi_{ij}) = \gamma_{0,i} + \gamma_{1,i} \tilde{a}_j.$$

This yields voxel-specific estimates $\hat{\gamma}_{0,i}$ and $\hat{\gamma}_{1,i}$, as well as predicted zero-inflation probabilities $\hat{\pi}_{ij}$. In the second step, we fit an ordinary least squares regression to the non-zero observations at each voxel to estimate the conditional mean. For those subjects where $Y_{ij} > 0$, we model the observed values as:

$$Y_{ij} = \beta_{0,i} + \beta_{1,i} \tilde{a}_j + \beta_{2,i} \tilde{a}_j^2 + \varepsilon_{ij}, \quad \varepsilon_{ij} \sim \mathcal{N}(0, \sigma_i^2).$$

From this, we obtain estimates $\hat{\boldsymbol{\beta}}_i = (\hat{\beta}_{0,i}, \hat{\beta}_{1,i}, \hat{\beta}_{2,i})$, the predicted conditional means $\hat{\mu}_{ij}$, and the residual standard deviation $\hat{\sigma}_i$. An approximate log-likelihood is then computed for each voxel using both the zero and non-zero components:

$$\log L_i \approx \sum_{j: Y_{ij} = 0} \log(\hat{\pi}_{ij} + \varepsilon) + \sum_{j: Y_{ij} > 0} \left[\log(1 - \hat{\pi}_{ij} + \varepsilon) + \log\left(\phi(Y_{ij}; \hat{\mu}_{ij}, \hat{\sigma}_i^2) + \varepsilon \right) \right],$$

where $\phi(\cdot; \mu, \sigma^2)$ is the standard Gaussian density and ε is a small positive constant (e.g., 10^{-10}) added for numerical stability. This procedure returns, for each voxel, the estimated regression coefficients $\hat{\boldsymbol{\beta}}_i$ and $\hat{\boldsymbol{\gamma}}_i$, the standard deviation $\hat{\sigma}_i$, predicted conditional means $\hat{\mu}_{ij}$, predicted zero-inflation probabilities $\hat{\pi}_{ij}$, and the approximate log-likelihood $\log L_i$. By separating the estimation of the two model components, the procedure remains robust, computationally efficient, and scalable to high-dimensional cortical data.

15 Estimation of Lifespan Trajectories of Conduction Delays

To characterize the lifespan trajectory of cortical conduction delays, we used the F-TRACT atlas as a structural reference. This atlas provides an empirically derived matrix of interareal conduction delays averaged over adults (> 15 years) as reported by Lemaréchal et al. [13], and served as the baseline constraint for the generative ξ–αNET model.

For each participant j, the subject-specific delay matrix $\mathbf{D}^{(j)}$ was estimated as a scaled version of the F-TRACT baseline:

$$\mathbf{D}^{(j)} = w_D^{(j)} \bar{\mathbf{D}}, \tag{7}$$

where $\bar{\mathbf{D}} \in \mathbb{R}^{N_v \times N_v}$ denotes the reference delay matrix and $w_D^{(j)}$ is a dimensionless scaling factor capturing individual variation across the lifespan. The scaling parameter was constrained within a physiologically plausible range:

$$|w_D^{(j)} - 1| < \frac{\Delta_\tau}{\mu_\tau}, \tag{8}$$

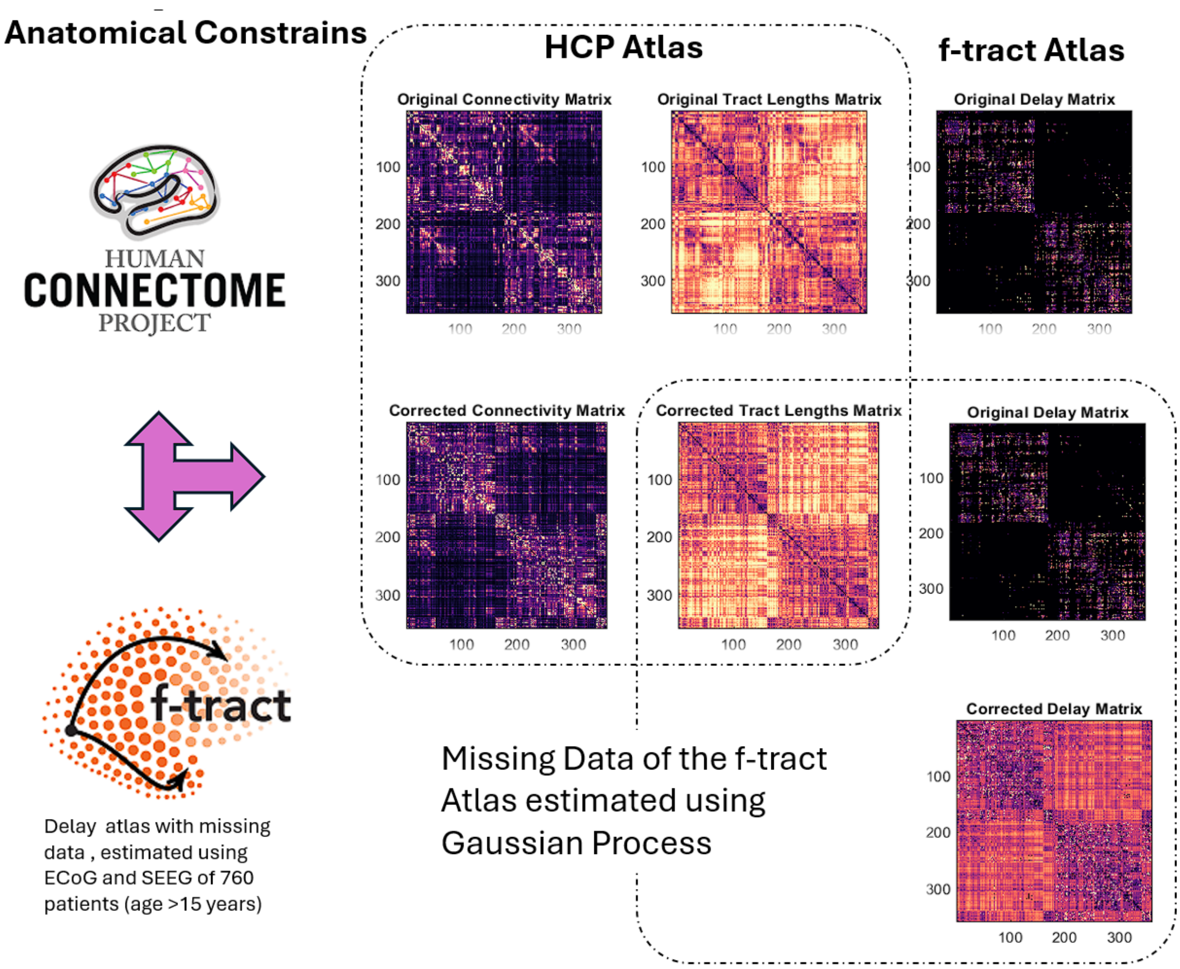

Figure 3: The baseline structural connectivity data used in this paper originates from the Human Connectome Project (HCP) [10] was estimated using diffusion MRI (dMRI) [21]. The baseline conduction delays atlas, which contains missing data, was obtained from the F-TRACT Consortium using cortico-cortical evoked potentials (CCEP) [13] Both atlases were coregistered to the HCP-MMP1 parcellation. Multiple preprocessing pipelines were applied to integrate structural connectivity and conduction delay data at a high source resolution of 8,003 voxels. Missing conduction delay values between neurotracts were interpolated using Gaussian Process modeling.

with $\Delta_\tau = 5.5$ ms and $\mu_\tau = 9.5$ ms corresponding to the median absolute deviation and median delay reported by Lemaréchal et al. [13]. Bayesian model inversion of ξ–αNET across the full HarMNqEEG dataset ($N = 1965$) yielded individual estimates of $w_D^{(j)}$, from which the average cortical conduction delay was derived as:

$$\langle \mathbf{D}^{(j)} \rangle = w_D^{(j)} \, \mu_\tau. \tag{9}$$

Finally, the electrophysiological *myelination proxy* was defined as $1/\langle \mathbf{D}^{(j)} \rangle^2$ and analyzed as a function of chronological age a_j. Lifespan trajectories were fitted using eight piecewise cubic B-spline basis functions, with optimal smoothing parameters selected via Akaike Information Criterion (AIC). Robust quadratic regression was further used to quantify the significance of curvature (p_{quad}) in the resulting trajectory.

16 Gaussian-Process Estimation of ROI-Level Conduction Delays and Projection to Voxel Space

The F-TRACT conduction-delay atlas is supplied not at the voxel level but in HCP–MMP 1.0 parcel space [13]; its entries form a sparse ROI-by-ROI matrix

$$\mathbf{D}_{\text{ROI}} \in \mathbb{R}^{N_r \times N_r}, \qquad N_r = 360,$$

with many $D_{\text{ROI},ij}$ unobserved. A complete companion matrix of inter-areal tract lengths,

$$\mathbf{L}_{\text{ROI}} \in \mathbb{R}^{N_r \times N_r},$$

is available from dMRI tractography [21, 22]. We impute the missing delays directly in this 360 $\times$ 360 ROI space. For every observed pair we set $x_{ij} = \log L_{\text{ROI},ij}$ and $y_{ij} = \log D_{\text{ROI},ij}$ and fit a Gaussian-process regression model

$$y_{ij} \sim \mathcal{N}\big(f(x_{ij}), \sigma_y^2\big), \qquad f \sim \mathcal{GP}\big(\mu, K\big), \quad K(x, x') = \sigma_f^2 \exp\big[-(x - x')^2/(2\ell^2)\big].$$

Hyperparameters $\{\mu, \sigma_f^2, \ell, \sigma_y^2\}$ are selected by maximising the marginal likelihood over the observed entries. The GP posterior mean $\mu_*(x)$ provides a smooth mapping from tract length to expected delay, and we set

$$\widehat{D}_{\text{ROI},ij} = \begin{cases} D_{\text{ROI},ij}, & \text{if observed,} \\ \exp\big[\mu_*(\log L_{\text{ROI},ij})\big], & \text{otherwise.} \end{cases}$$

To obtain voxel-level priors, every voxel inherits the delay value of its parent ROI. Let $\mathcal{V}_r$ denote the index set of voxels belonging to ROI r. The voxel-by-voxel delay matrix is defined as

$$\widehat{D}_{uv} = \widehat{D}_{\text{ROI},\, r(u)\, r(v)}, \qquad u \in \mathcal{V}_{r(u)},\ v \in \mathcal{V}_{r(v)},$$

and the same replication procedure converts $\mathbf{L}_{\text{ROI}}$ to a voxel-level length matrix $\mathbf{L}$. The resulting fully populated matrices $\widehat{\mathbf{D}}$ and $\mathbf{L}$ are the structural priors used in the inversion of the ξ–αNET model.

17 Average Reference Transformation

To standardize all input cross-spectral matrices under a common reference scheme, each subject's cross-spectrum $\mathbf{S}_\omega^{(j)} \in \mathbb{C}^{N_c \times N_c}$ was transformed to the average reference montage using the following operation:

$$\tilde{\mathbf{S}}_\omega^{(j)} = \Big[\mathbf{H}\mathbf{S}_\omega^{(j)}\mathbf{H}^\top\Big]_{1:(N_c-1),\, 1:(N_c-1)},$$

where the centering matrix is defined as

$$\mathbf{H} = \mathbf{I}_{N_c} - \frac{1}{N_c}\mathbf{1}_{N_c}\mathbf{1}_{N_c}^\top.$$

Here, $\mathbf{I}_{N_c}$ denotes the identity matrix and $\mathbf{1}_{N_c}$ is a column vector of ones. This average reference transformation removes the common mode across all channels and introduces a linear dependency among electrodes—a known property of unipolar referencing. To preserve full rank, we removed the redundant channel Pz, reducing the number of channels from $N_c = 19$ to $N_c = 18$. The resulting matrix $\tilde{\mathbf{S}}_\omega^{(j)}$ was used as the input for all subsequent analyses in the Xi–AlphaNET framework.

18 Global-Scale Factor (GSF) Correction

EEG cross-spectra often differ in global amplitude due to non-neural sources of variance such as electrode impedance, amplifier gain, scalp and hair thickness, and other recording-specific factors. To mitigate this variability, we applied a global-scale factor (GSF) correction following the method introduced by Hernández et al. (1994). Let the EEG signal at channel c, epoch e, and time t for subject j be modeled as:

$$v_{e,c}^{(j)}(t) = \gamma_j\, \beta_{e,c}^{(j)}(t),$$

where $\gamma_j > 0$ is the global scale factor and $\beta_{e,c}^{(j)}(t)$ is the scale-independent signal. Let $\mathbf{S}_\omega^{(j)}$ denote the cross-spectrum matrix for subject j at frequency ω. Its eigendecomposition is given by:

$$\mathbf{S}_\omega^{(j)} = \mathbf{\Gamma}_\omega^{(j)}\mathbf{D}_\omega^{(j)}\left(\mathbf{\Gamma}_\omega^{(j)}\right)^{\mathrm{H}},$$

where $\mathbf{\Gamma}_\omega^{(j)}$ contains the eigenvectors and $\mathbf{D}_\omega^{(j)}$ is the diagonal matrix of eigenvalues. Rescaling by γ_j, the log cross-spectrum becomes:

$$\log\left(\frac{\mathbf{S}_\omega^{(j)}}{\gamma_j^2}\right) = \log \mathbf{S}_\omega^{(j)} - \kappa_j \mathbf{I}, \quad \text{where } \kappa_j = 2\log\gamma_j.$$

The GSF contributes an additive shift to the diagonal of the log cross-spectrum. The maximum likelihood estimate of κ_j is:

$$\hat{\kappa}_j = \frac{1}{N_\omega N_c} \sum_{\omega} \sum_{c=1}^{N_c} \log \left(s_{c,c,\omega}^{(j)} \right),$$

where $s_{c,c,\omega}^{(j)}$ denotes the power (diagonal entry) at channel c and frequency ω. The GSF-corrected cross-spectrum is then:

$$\tilde{\mathbf{S}}_\omega^{(j)} = \frac{\mathbf{S}_\omega^{(j)}}{\exp(\hat{\kappa}_j)}.$$

This transformation ensures that the estimated spectral features—$A\xi$, $A\alpha$, and $F\alpha$—reflect physiologically meaningful differences and are not confounded by global amplitude variability.

19 Code and Data Reproducibility

19.1 Data Availability

Access through our institutional OneDrive `Xi-AlphaNET-DATA`,

19.2 How to reproduce the analysis of each figure of the paper

All programs required to fully reproduce the results presented in this paper are available in the GitHub repository `Xi-AlphaNET`, or upon reasonable request to the corresponding author. Most analyses employ parallel computing routines implemented in MATLAB; therefore, we recommend using MATLAB R2024a or later. Users should ensure that their system provides sufficient computational resources to support parallel execution. If resources are limited, the `parfor` loops can be manually replaced by standard `for` loops.

Before execution, users must modify the `json_path` and `dir_path` variables to point respectively to the `XIALPHANET.json` file generated by running Xi-AlphaNET on the HarMNqEEG dataset, and to the directory containing the corresponding unprocessed HarMNqEEG normative data. The MATLAB functions required to reproduce all figures are organized within the `+functions/+auxx/+Regressions` directory.

To reproduce **Figure 6**, download the required data, adjust the `json_path` variable in line 4 of the script, and run the function `delays_vs_age.m`.

To reproduce **Figure 3**, several programs must be executed, some of which rely on the DataViz toolbox available at `DataViz`. For panels (a)–(b), run `model_spatial_dispersion.m`, updating the path to `parameters.mat` with the corresponding leadfields located in the `structural` folder of the supplementary data. For panels (c)–(d), download the test–retest dataset and adjust the path in the `icc_vertex` function, which automatically produces these figures. Additional visualization of the test–retest analysis can be performed using the Xi-AlphaNET graphical interface following the guidelines on GitHub. Panels (e)–(h) can be reproduced by running the `neural_field.m` script.

To generate the probability atlases shown in **Figure 4 (a, e, c, g)**, use the `sexy_range_prob.m` script. To reproduce the analysis of average spectral Granger causality across the lifespan (**Figure 4 d, h**), run `effective_population_spectral_net.m`, which automatically computes Granger effective connectivity for each age group, evaluates spatial null models, and visualizes the results using `effective_spectral_net.m`. To obtain panels (i, j) and the average spectral Granger causality over the entire HarMNqEEG dataset (**Figure 4 b, f**), perform the permutation test of Granger causal asymmetry across the cortical hierarchy index using the `hierch_DAI` function. The circular plots summarizing significant connections in Yeo–7 networks are generated with the `circos_plot_test.m` function, adapted from `circos plot` by Zhaoxu Liu / slandarer (2025), available on MATLAB Central File Exchange: `circosplot`.

Finally, to reproduce **Figure 5**, run the `zi_plots.m` script, which employs the `fitZIG_random.m` function to fit the zero-inflated Gaussian (ZIG) model.

20 Laplace-Based Posterior Inference and Information-Geometric Identifiability

20.1 Laplace Approximation of Posterior Distribution & Fisher Information Matrix

We approximate the posterior distribution using a local Laplace approximation [12], which requires the computation of the maximum a posteriori (MAP) estimate of the model parameters for each subject j in the HarMNqEEG dataset, $\hat{\mathbf{x}}^{(j)}$, as well as the evaluation of the Fisher Information Matrix at the MAP estimate. The Fisher information is computed from the smooth log-likelihood using the standard expression $\mathcal{I}_{\mathrm{lik}}(\mathbf{x}) = \mathbb{E}\big[\nabla^{\dagger} f(\mathbf{x})\,\nabla f(\mathbf{x})\big]$, where f and ∇f denote the ξ–αNET log-likelihood and its score function, respectively (see Supplementary Section S4) [1]. Under these conditions, the posterior distribution in the neighborhood of the MAP estimate is approximated by a multivariate Gaussian distribution.

$$p(\mathbf{x}|\mathbf{S}^{(j)}) \approx \mathcal{N}(\mathbf{x}|(\hat{\mathbf{x}}^{(j)}, \mathbf{\Sigma}_{\mathbf{x}}^{(j)}), \tag{10}$$

$$\mathbf{\Sigma}_{\mathbf{x}}^{(j)} = \Big(\mathcal{I}_{\mathsf{lik}}(\hat{\mathbf{x}}^{(j)}) + H^{\mathsf{loc}}_{\mathsf{prior}}(\hat{\mathbf{x}}^{(j)})\Big)^{-1}. \tag{11}$$

Here, $\mathcal{I}_{\mathsf{lik}}$ denotes the Fisher information matrix derived from the smooth likelihood term. After some straightforward algebraic derivation, it can be shown that the Fisher Information matrix, given a point in parameter space $\mathbf{x}$ has the following closed formula

$$\mathcal{I}_{\mathsf{lik}}(\mathbf{x}) = \sum_{\omega} (\nabla_{\mathbf{x}} \mathbf{R}_{\omega})^{\dagger} (\mathbf{R}_{\omega}^{-1} \otimes \mathbf{R}_{\omega}^{-1}) \nabla_{\mathbf{x}} \mathbf{R}_{\omega}, \tag{12}$$

where $\mathbf{R}_{\omega} = \sigma_{\omega}^2 \mathbf{I}_{N_c} + \mathbf{T}_{\omega} \mathrm{diag}(\boldsymbol{\xi}_{\omega} + \boldsymbol{\alpha}_{\omega}) \mathbf{T}_{\omega}^{\dagger}$ tis the covaraince of the observed data. This expression highlights that the Fisher information depends exclusively on first-order variations of $\mathbf{R}_{\omega}$ all second-derivative terms vanish in expectation and therefore do not contribute. Geometrically, the Fisher information defines a Riemannian metric on the parameter space of the ξ–αNET model. The parameter vector $\mathbf{x}$ is mapped to the manifold of symmetric positive–definite matrices through the smooth embedding $\mathbf{x} \longmapsto \mathbf{R}_{\omega}(\mathbf{x})$, where $\mathbf{R}_{\omega}$ denotes the model-implied cross-spectral covariance at frequency ω. The tensor $\mathbf{R}_{\omega}^{-1} \otimes \mathbf{R}_{\omega}^{-1}$ corresponds to the canonical information metric on this manifold. The Fisher information matrix, therefore, arises as the pullback of this metric through the parameter-to-covariance mapping, endowing the parameter space with an intrinsic notion of distance and curvature induced by the likelihood. Under this interpretation, infinitesimal perturbations of the parameters are measured according to how strongly they deform the covariance structure $\mathbf{R}_{\omega}$. Directions in parameter space along which $\mathbf{R}_{\omega}$ varies weakly correspond to low curvature of the log-likelihood and thus high posterior uncertainty, whereas directions that induce great changes in $\mathbf{R}_{\omega}$ correspond to high curvature and low uncertainty. In this sense, the Fisher information provides a principled geometric measure of identifiability and sensitivity for each spectral parameter [1]. On the other hand, $H^{\mathsf{loc}}_{\mathsf{prior}}$ denotes a local quadratic approximation of the prior restricted to the active set of parameters. The resulting covariance matrix $\mathbf{\Sigma}$ provides closed-form posterior variances and credibility intervals for all ξ–αNET spectral parameters.

20.2 Impact of Signal and Channel Quality on the Xi-AlphaNET estimation

First, it is important to emphasize that the application of ξ–αNET, as with any EEG inverse solution, requires standard preprocessing steps to remove artifacts and non-neural contamination. This includes established procedures such as bad-channel detection, artifact rejection, and independent component analysis, for example using widely adopted toolboxes such as EEGLAB. These steps are necessary to ensure that the data entering the generative model satisfy the basic assumptions of stationarity and approximate Gaussianity.

Beyond this mandatory preprocessing, the ξ–αNET framework allows the impact of signal and channel quality on parameter estimation to be quantified in a principled and transparent manner. This follows directly from the closed-form expression of the Fisher information matrix (Eq. 12). In particular, in regimes where sensor noise contributes non-negligibly to the observed covariance, a first-order Neumann expansion yields

$$\mathcal{I}_{ij} \approx \mathcal{I}_{ij}^{(0)} - \sum_{\omega} \sum_{c=1}^{N_c} \sigma_{\omega,c}^2 \, \mathcal{W}_{ij}^{(\omega,c)},$$

where $\mathcal{I}_{ij}^{(0)}$ denotes the noise-free Fisher information and $\sigma_{\omega,c}^2$ is the noise variance of sensor c at frequency ω. This expression explicitly states that each sensor contributes an additive, negative correction to the Fisher information, which is linear in its noise variance. Consequently, channels with elevated noise or poor signal

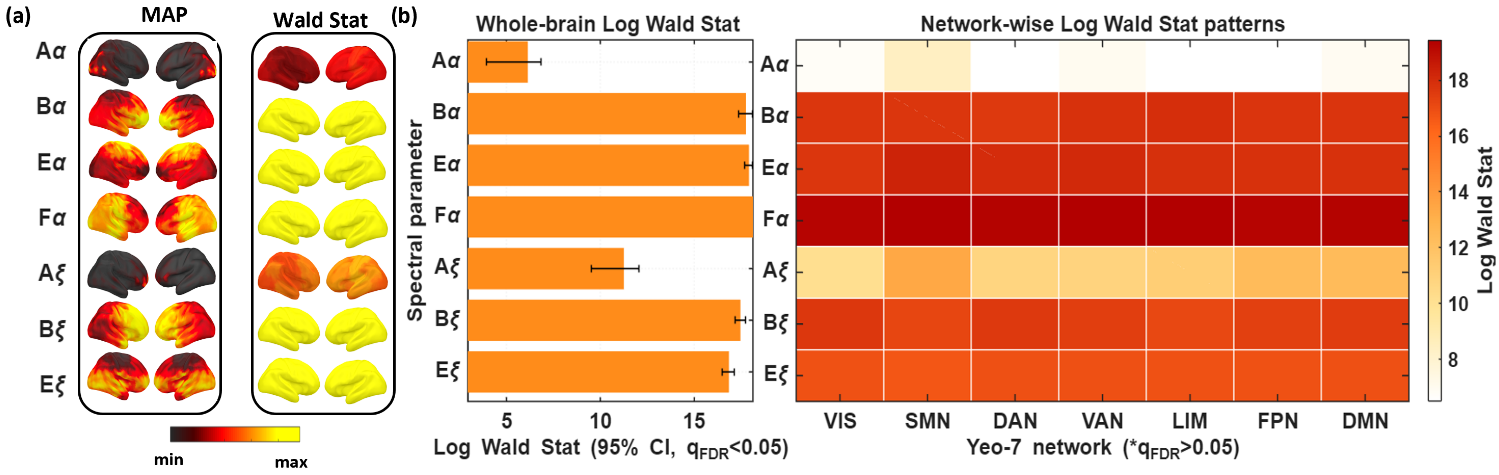

Figure 4: Bayesian Identifiability and Information-Geometric Characterization of ξ–αNET Spectral Parameters. (a) Cortical topography of Maximum A Posteriori (MAP) estimates and Wald identifiability. The left panels illustrate the MAP estimates for the ξ–αNET spectral parameters averaged across the HarMNqEEG cohort. The right panels display the corresponding Wald statistics ($\mathbf{W}$), which serve as a formal measure of parameter identifiability. High Wald values indicate regions where parameters are tightly constrained by the data, corresponding to high curvature of the log-likelihood (or log-posterior). This curvature is mathematically captured by the Fisher Information Matrix (FIM), which facilitates a local quadratic Laplace approximation of the prior restricted to the active set of parameters. (b) Population-level precision and network-wise distribution. The central bar plot displays the whole-brain log Wald statistics (mean $\pm$ 95% CI), where each statistic is calculated as the squared ratio of the MAP estimate to its posterior variance: $\mathbf{W}_{\mathbf{x}}^{(j)} = (\hat{\mathbf{x}}^{(j)})^2 / \operatorname{diag}(\mathbf{\Sigma}_x^{(j)})$. The posterior covariance $\mathbf{\Sigma}$ is derived from the inversion of the FIM, endowing the parameter space with an intrinsic information-geometric metric induced by the parameter-to-covariance mapping. The right heatmap resolves these identifiability metrics across the Yeo-7NET. Statistical significance was assessed using nonparametric permutation testing against the null hypothesis $H_0 : W = 0$, with multiple comparisons controlled via False Discovery Rate (FDR) correction. Overall, this analysis reveals that parameter estimation is tightly constrained by the generative model and its structural priors. Locally, around the MAP estimate, the parameters exhibit high curvature across the majority of regions of interest (ROIs), indicating robust identifiability and stable posterior solutions at the population level.

quality locally flatten the likelihood, reducing curvature along parameter directions that depend on those sensors. Within the Laplace approximation, this reduction in curvature translates directly into inflated posterior variances, resulting in wider credibility intervals for the affected parameters. Importantly, this mechanism provides an automatic and principled down-weighting of unreliable channels without requiring their explicit exclusion. Poor-quality sensors contribute less information to the inference, while high-quality channels preserve parameter identifiability. As a result, ξ–αNET naturally propagates channel-level reliability into uncertainty estimates, enabling a quantitative assessment of how signal and channel quality affect the robustness of the inferred spectral parameters.

20.3 Posterior Uncertainty and Parameter Identifiability

To summarize and characterize the posterior distributions estimated with Xi-AlphaNET on the HarMNqEEG dataset [14], we projected the maximum a posteriori (MAP) estimates obtained at the cortical surface level (8,003 vertices) onto the HCP-MMP1 parcellation (360 regions per hemisphere) [10]. This dimensionality reduction is required because full posterior characterization would involve inversion of the Fisher Information Matrix for nearly 2,000 subjects to estimate the posterior covariance $\mathbf{\Sigma}_{\mathbf{x}}^{(j)}$, which is computationally prohibitive at the vertex level. Mapping the MAP estimates to regions of interest (ROIs) renders this computation tractable while providing a valid approximation of posterior uncertainty. Posterior uncertainty was summarized using the Wald statistic [16] at each ROI,

$$\mathbf{W}_{\mathbf{x}}^{(j)} = \frac{\left(\hat{\mathbf{x}}^{(j)}\right)^2}{\operatorname{diag}\left(\mathbf{\Sigma}_{\mathbf{x}}^{(j)}\right)}, \tag{13}$$

where squaring and division are performed element-wise. Unlike confidence intervals or relative uncertainty measures, the Wald statistic provides a stable quantification of the MAP estimate relative to its uncertainty,

which is particularly advantageous in sparse regimes such as Xi-AlphaNET, where MAP-normalized confidence ratios may be ill-defined. In the Bayesian setting, the Wald statistic reflects parameter identifiability: large values indicate parameters that are both non-zero and tightly constrained by the data, corresponding to high curvature of the log-posterior around the MAP estimate. From an information-geometric perspective, this curvature reflects strong constraints imposed by the generative model and its structural priors, whereas low Wald values indicate weakly constrained, flat posterior directions [16]. Wald statistics were computed for each subject and mapped to the HCP-MMP1 parcellation. Population-level effects were obtained by averaging across subjects. For each ROI r, grouped according to the Yeo-7 networks [26], we tested the null hypothesis $H_0 : W_{x,r}^{(j)} = 0$ using nonparametric permutation testing, with multiple comparisons controlled by false discovery rate (FDR) correction. Overall, this analysis separates parameter magnitude, captured by MAP estimates, from parameter identifiability, captured by the Wald statistic, enabling the robust identification of cortical regions and networks in which ξ–αNET yields stable and well-constrained posterior solutions at the population level.

The results of this analysis are summarized in Fig. 4. The figure demonstrates that, across all spectral parameters of the ξ–αNET model, the Wald statistics are consistently elevated at the population level, indicating that the corresponding posterior distributions are tightly constrained by the data and the generative model. After correction for multiple comparisons using false discovery rate (FDR), all parameters remain significant across the Yeo-7NET. Although the alpha-process amplitude parameter ($A\alpha$) exhibits comparatively lower Wald values, it nonetheless remains significantly above zero, reflecting robust—albeit weaker—posterior identifiability relative to the other parameters. Overall, these results confirm that the ξ–αNET inversion yields stable and well-identified posterior solutions across cortical regions and large-scale functional networks.

21 Individual-Level ξ–αNET Inference and Clinical Translation: A Parkinson's Disease Case Study

We demonstrate the utility of the ξ-αNET model in diagnosing and extracting biomarkers for Parkinson's disease.

For this purpose, we applied the ξ-αNET model to the Parkinson's disease dataset from the NeuroEPO clinical project conducted in Cuba, which had already been preprocessed to eliminate artifacts [3]. The dataset includes subjects at various stages of Parkinson's disease, with measurements taken at two different time points: T0, prior to the administration of different doses of the NeuroEPO drug, and T1, after the administration. The subjects at T0 had ages ranging from 41 to 72 years. We specifically used the T0 EEG data, which includes subjects with Parkinson's disease before any drug intervention, and applied ξ-αNET to estimate the source spectral and structural parameters. After performing Bayesian inversion of the NeuroEPO data, we compared the results for the same age group with those from the ξ-αNET inversion of the HarMNqEEG data (N = 264 of 1965), which represent healthy controls at rest.

Figure 5 (a) presents the scatter plot of the cortical average delay and spectral parameters for the control subjects from HarMNqEEG (N = 264, gray points) against the Parkinson's disease group (N = 34, red points). Visual inspection reveals that the Parkinson's disease group exhibits increased conduction delays, higher alpha and ξ-amplitudes [1], and a slowing of the peak alpha frequency (PAF) relative to the control group. This suggests an increase in the synchronization of slow alpha rhythms in the Parkinson's group.

Figure 5 (b) shows the network-wise differences in the distributions of spectral parameters between the control and Parkinson's groups, represented for each network in the Yeo-7 network parcellation using boxplots. Group differences were quantified using the Mann–Whitney U-test[2], with p-values corrected using the False Discovery Rate (FDR). Asterisks indicate significant effects ($^*q_{FDR} < 0.05$). As shown in the figure, across all networks, the Parkinson's group exhibits an increase in alpha amplitude and a decrease in peak frequency, accompanied by a narrowing of the bandwidth. Indicating a robust increase in slow alpha synchronization. No significant changes were detected for the rate of decay of the frequency (or the exponent) of the alpha rhythm after FDR correction. These findings are consistent with previous literature that describes the slowing of alpha rhythms in Parkinson's disease [27, 24]. On the other hand, for the ξ-process (the aperiodic component), we observed more significant differences across all networks. The amplitude of the ξ-rhythm increased, while the bandwidth expanded and the exponent decayed significantly in almost all networks. This suggests a flattening of the

[1] The amplitudes of the ξ-αNET model should not be confused with the power of these rhythms. The former refers to the probability associated with the spectral components, while the latter refers to the area under the power spectrum curve.

[2] We use this test because the assumptions of normality are not met for the amplitudes and some other parameters.

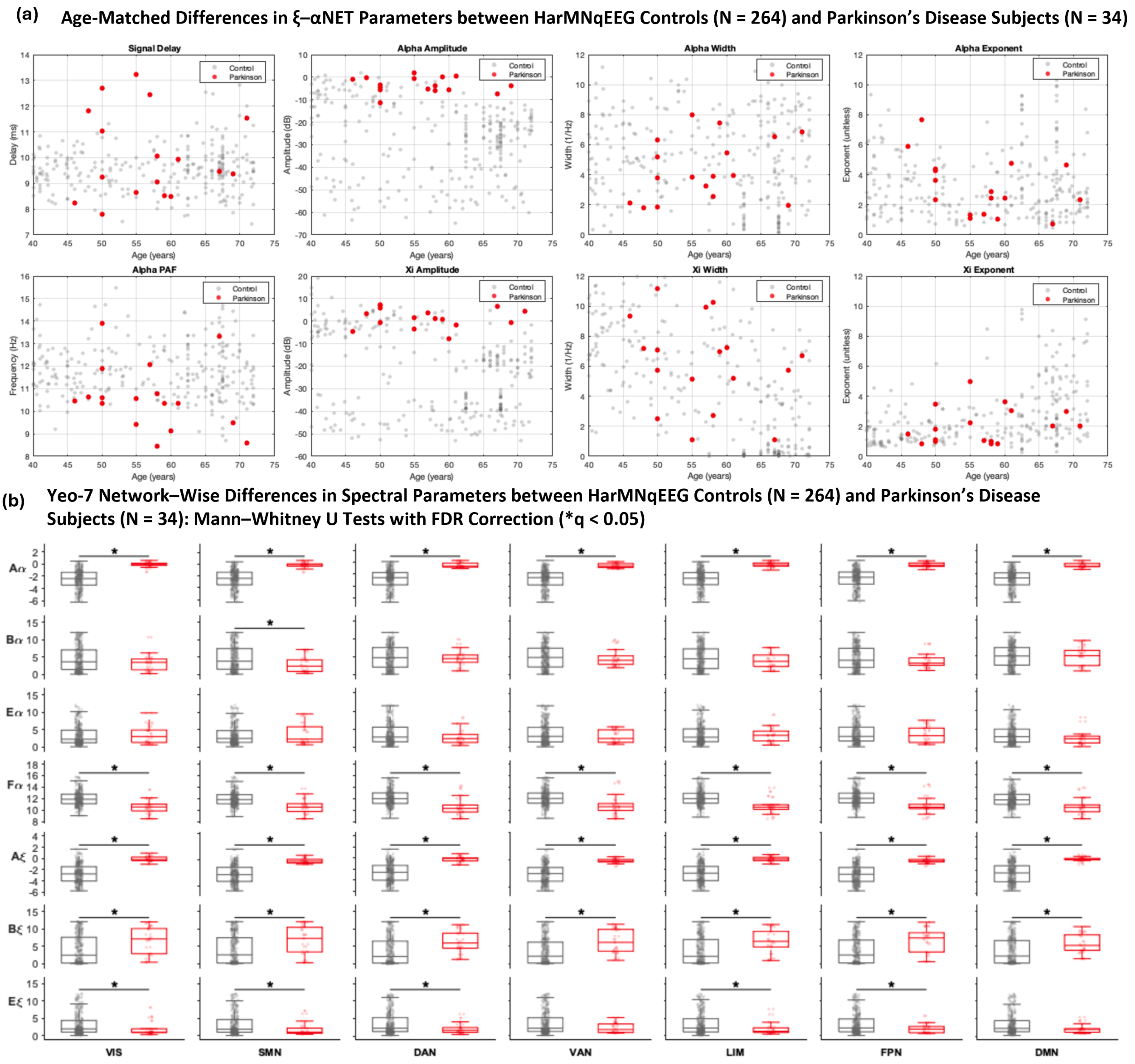

Figure 5: **Age-matched alterations of ξ–αNET spectral parameters in Parkinson's disease. (a)** Subject-level distributions of ξ–αNET parameters plotted as a function of age for HarMNqEEG healthy controls (gray; $N = 264$) and Parkinson's disease subjects (red; $N = 34$). HarMNqEEG controls were explicitly selected from the same age range as the Parkinson's disease cohort, ensuring strict age matching between groups. Shown are signal conduction delay, α-process amplitude, bandwidth, exponent, and peak alpha frequency ($F\alpha$), together with the corresponding ξ-process amplitude, bandwidth, and exponent. Despite age matching, Parkinson's disease subjects exhibit systematic deviations from the normative control distribution, most prominently a shift toward lower $F\alpha$ accompanied by relatively enhanced low-α amplitude, consistent with slow alpha synchronization, alongside marked alterations in ξ-process parameters. **(b)** Yeo-7 network–wise comparisons of ξ–αNET parameters between groups. Boxplots summarize regional distributions for controls (gray) and Parkinson's disease subjects (red). Group differences were assessed using Mann–Whitney U tests with false discovery rate correction; asterisks denote significant effects ($q < 0.05$). Across all networks, the α process exhibits a robust increase in amplitude, accompanied by a systematic reduction in alpha frequency, indicating enhanced and more narrowly tuned *slow alpha synchronization*. In contrast, the aperiodic ξ process exhibits an increase in amplitude, an expansion of bandwidth, and a decay of the spectral exponent, reflecting a broadband elevation of aperiodic activity and a flattening of the background spectrum. These complementary effects highlight a coordinated reorganization of oscillatory (α) and aperiodic (ξ) dynamics across large-scale cortical networks in Parkinson's disease.

background aperiodic activity across the cortical networks in Parkinson's disease.

These results suggest that the ξ-αNET parameters can be utilized to identify biomarkers for Parkinson's disease, with immediate implications for both clinical and research applications. This example illustrates how individual-level spectral parameters can be utilized in clinical research. In future work, we aim to expand on this analysis to create normative datasets that will enable the identification of biomarkers for various diseases, including, but not limited to, Parkinson's disease.

References

[1] Shun-ichi Amari. Information geometry and its applications. *Journal of Mathematical Psychology*, 49:101–102, 2005.

[2] Amir Beck and Marc Teboulle. A fast iterative shrinkage-thresholding algorithm for linear inverse problems. *SIAM Journal on Imaging Sciences*, 2(1):183–202.

[3] Maria L Bringas Vega, Ivonne Pedroso Ibáñez, Fuleah A Razzaq, Min Zhang, Lilia Morales Chacón, Peng Ren, Lidice Galan Garcia, Peng Gan, Trinidad Virues Alba, Carlos Lopez Naranjo, et al. The effect of neuroepo on cognition in parkinson's disease patients is mediated by electroencephalogram source activity. *Frontiers in Neuroscience*, 16:841428, 2022.

[4] Joshua B Burt, Murat Demirtaș, William J Eckner, Natasha M Navejar, Jie Lisa Ji, William J Martin, Alberto Bernacchia, Alan Anticevic, and John D Murray. Hierarchy of transcriptomic specialization across human cortex captured by structural neuroimaging topography. *Nature neuroscience*, 21(9):1251–1259, 2018.

[5] Mukeshwar Dhamala, Govindan Rangarajan, and Mingzhou Ding. Analyzing information flow in brain networks with nonparametric granger causality. *Neuroimage*, 41(2):354–362, 2008.

[6] Lihong Ding, Wei Duan, Yulin Wang, and Xu Lei. Test–retest reproducibility comparison in resting and mental task states: A sensor- and source-level eeg spectral analysis. *International Journal of Psychophysiology*, 173:20–28, 2022.

[7] Jianqing Fan. *Local polynomial modelling and its applications: monographs on statistics and applied probability 66*. Routledge, 2018.

[8] G. Fort, L. Risser, Y. Atchadé, and E. Moulines. Stochastic fista algorithms: So fast ? In *2018 IEEE Statistical Signal Processing Workshop (SSP)*, pages 796–800, 2018.

[9] Jerome Friedman, Trevor Hastie, and Robert Tibshirani. A note on the group lasso and a sparse group lasso. *arXiv preprint arXiv:1001.0736*, 2010.

[10] Matthew F. Glasser, Timothy S. Coalson, Emma C. Robinson, Carl D. Hacker, John Harwell, Essa Yacoub, Kamil Ugurbil, Jesper Andersson, Christian F. Beckmann, and Mark Jenkinson. A multi-modal parcellation of human cerebral cortex. *Nature*, 536(7615):171–178. Publisher: Nature Publishing Group UK London.

[11] Tanguy Hedrich, Giovanni Pellegrino, Eliane Kobayashi, Jean-Marc Lina, and Christophe Grova. Comparison of the spatial resolution of source imaging techniques in high-density eeg and meg. *NeuroImage*, 157:531–544, 2017.

[12] Robert Kass, Luke Tierney, and Joseph Kadane. Laplace's method in bayesian analysis. *Contemporary Mathematics*, pages 89–135, 1991.

[13] Jean-Didier Lemaréchal, Maciej Jedynak, Lena Trebaul, Anthony Boyer, François Tadel, Manik Bhattacharjee, Pierre Deman, Viateur Tuyisenge, Leila Ayoubian, Etienne Hugues, Blandine Chanteloup-Forêt, Carole Saubat, Raouf Zouglech, Gina Catalina Reyes Mejia, Sébastien Tourbier, Patric Hagmann, Claude Adam, Carmen Barba, Fabrice Bartolomei, Thomas Blauwblomme, Jonathan Curot, François Dubeau, Stefano Francione, Mercedes Garcés, Edouard Hirsch, Elizabeth Landré, Sinclair Liu, Louis Maillard, Eeva-Liisa Metsähonkala, Ioana Mindruta, Anca Nica, Martin Pail, Ana Maria Petrescu, Sylvain Rheims, Rodrigo Rocamora, Andreas Schulze-Bonhage, William Szurhaj, Delphine Taussig, Antonio Valentin, Haixiang Wang, Philippe Kahane, Nathalie George, Olivier David, F-TRACT consortium, Claude Adam, Vincent Navarro, Arnaud Biraben, Anca Nica, Dominique Menard, Milan Brazdil, Robert Kuba, Jitka Kočvarová, Martin Pail, Irena Doležalová, François Dubeau, Jean Gotman, Philippe Ryvlin, Jean Isnard, Hélène

Catenoix, Alexandra Montavont, Sylvain Rheims, Fabrice Bartolomei, Agnès Trébuchon, Aileen McGonigal, Wenjing Zhou, Haixiang Wang, Sinclair Liu, Zhang Wei, Zhu Dan, Guo Qiang, Hu Xiangshu, Li Hua, Hua Gang, Wang Wensheng, Mei Xi, Feng Yigang, Rima Nabbout, Marie Bourgeois, Anna Kaminska, Thomas Blauwblomme, Mercedes Garcés, Antonio Valentin, Rinki Singh, Liisa Metsähonkala, Eija Gaily, Leena Lauronen, Maria Peltola, Francine Chassoux, Elizabeth Landré, Philippe Derambure, William Szurhaj, Maxime Chochois, Edouard Hirsch, Maria Paola Valenti, Julia Scholly, Luc Valton, Marie Denuelle, Jonathan Curot, Rodrigo Rocamora, Alessandro Principe, Miguel Ley, Ioana Mindruta, Andrei Barborica, Stefano Francione, Roberto Mai, Lino Nobili, Ivana Sartori, Laura Tassi, Louis Maillard, Jean-Pierre Vignal, Jacques Jonas, Louise Tyvaert, Mathilde Chipaux, Delphine Taussig, Philippe Kahane, Lorella Minotti, Anne-Sophie Job, Véronique Michel, Marie De Montaudoin, Jérôme Aupy, Viviane Bouilleret, Ana Maria Petrescu, Pascal Masnou, Claire Dussaule, Marion Quirins, Delphine Taussig, Carmen Barba, Renzo Guerrini, Matteo Lenge, and Elisa Nacci. A brain atlas of axonal and synaptic delays based on modelling of cortico-cortical evoked potentials. *Brain*, 145(5):1653–1667.

[14] Min Li, Ying Wang, Carlos Lopez-Naranjo, Shiang Hu, Ronaldo César García Reyes, Deirel Paz-Linares, Ariosky Areces-Gonzalez, Aini Ismafairus Abd Hamid, Alan C Evans, Alexander N Savostyanov, et al. Harmonized-multinational qeeg norms (harmnqeeg). *NeuroImage*, 256:119190, 2022.

[15] Nian Li, Jiaxin Yang, Chao Long, and Xu Lei. Test–retest reliability of eeg aperiodic components in resting and mental task states. *Brain Topography*, 37(6):961–971, 2024.

[16] Yang Liu, Youjin Sung, Jonathan P Williams, and Jan Hannig. Calibrating bayesian inference. *arXiv preprint arXiv:2510.27144*, 2025.

[17] Georgios Michalareas, Julien Vezoli, Stan Van Pelt, Jan-Mathijs Schoffelen, Henry Kennedy, and Pascal Fries. Alpha-beta and gamma rhythms subserve feedback and feedforward influences among human visual cortical areas. *Neuron*, 89(2):384–397, 2016.

[18] Dung A. Nguyen-Danse, Shobana Singaravelu, Léa A. S. Chauvigné, Anaïs Mottaz, Leslie Allaman, and Adrian G. Guggisberg. Feasibility of reconstructing source functional connectivity with low-density eeg. *Brain Topography*, 34(6):709–719, 2021.

[19] Roberto D Pascual-Marqui, Kieko Kochi, and Toshihiko Kinoshita. Cortical xi-alpha model for resting state electric neuronal activity. *arXiv preprint arXiv:2212.13571*, 2022.

[20] Roberto D. Pascual-marqui, Pedro A. Valdes-sosa, and Alfredo Alvarez-amador. A parametric model for multichannel EEG spectra. *International Journal of Neuroscience*, 40(1):89–99.

[21] Burke Q. Rosen and Eric Halgren. An estimation of the absolute number of axons indicates that human cortical areas are sparsely connected. *PLOS Biology*, 20(3):e3001575.

[22] Burke Q. Rosen and Eric Halgren. A whole-cortex probabilistic diffusion tractography connectome. *eNeuro*, 8(1). Publisher: Society for Neuroscience Section: Research Article: New Research.

[23] Patrick E Shrout and Joseph L Fleiss. Intraclass correlations: uses in assessing rater reliability. *Psychological bulletin*, 86(2):420, 1979.

[24] Raija Soikkeli, Juhani Partanen, Hilkka Soininen, Ari Pääkkönen, and Paavo Riekkinen Sr. Slowing of eeg in parkinson's disease. *Electroencephalography and clinical neurophysiology*, 79(3):159–165, 1991.

[25] Qing Wang, Pedro A. Valdés-Hernández, Deirel Paz-Linares, Jorge Bosch-Bayard, Naoya Oosugi, Misako Komatsu, Naotaka Fujii, and Pedro A. Valdés-Sosa. Eecog-comp: An open source platform for concurrent eeg/ecog comparisons—applications to connectivity studies. *Brain Topography*, 32(4):550–568, 2019.

[26] BT Thomas Yeo, Fenna M Krienen, Jorge Sepulcre, Mert R Sabuncu, Danial Lashkari, Marisa Hollinshead, Joshua L Roffman, Jordan W Smoller, Lilla Zöllei, Jonathan R Polimeni, et al. The organization of the human cerebral cortex estimated by intrinsic functional connectivity. *Journal of neurophysiology*, 2011.

[27] Yuqing Zhao, Jiayu Cai, Jian Song, Haoran Shi, Weicheng Kong, Xinlei Li, Wei Wei, and Xiehua Xue. Peak alpha frequency and alpha power spectral density as vulnerability markers of cognitive impairment in parkinson's disease: an exploratory eeg study. *Frontiers in Neuroscience*, 19:1575815, 2025.
